# Deciphering *cis*-regulatory elements using REgulamentary

**DOI:** 10.1093/bioadv/vbag079

**Published:** 2026-03-20

**Authors:** Simone G Riva, Edward Sanders, Emily Georgiades, Samvida S Venkatesh, Martin Sergeant, Emine Ravza Gür, Jennifer C Herrmann, Matthew Baxter, Jim R Hughes

**Affiliations:** MRC Molecular Haematology Unit, MRC Weatherall Institute of Molecular Medicine, University of Oxford, Oxford, OX3 9DS, United Kingdom; MRC WIMM Centre for Computational Biology, MRC Weatherall Institute of Molecular Medicine, University of Oxford, Oxford, OX3 9DS, United Kingdom; MRC Molecular Haematology Unit, MRC Weatherall Institute of Molecular Medicine, University of Oxford, Oxford, OX3 9DS, United Kingdom; MRC WIMM Centre for Computational Biology, MRC Weatherall Institute of Molecular Medicine, University of Oxford, Oxford, OX3 9DS, United Kingdom; MRC Molecular Haematology Unit, MRC Weatherall Institute of Molecular Medicine, University of Oxford, Oxford, OX3 9DS, United Kingdom; MRC WIMM Centre for Computational Biology, MRC Weatherall Institute of Molecular Medicine, University of Oxford, Oxford, OX3 9DS, United Kingdom; MRC WIMM Centre for Computational Biology, MRC Weatherall Institute of Molecular Medicine, University of Oxford, Oxford, OX3 9DS, United Kingdom; Department of Statistics, University of Oxford, Oxford, OX1 3LB, United Kingdom; MRC Molecular Haematology Unit, MRC Weatherall Institute of Molecular Medicine, University of Oxford, Oxford, OX3 9DS, United Kingdom; MRC WIMM Centre for Computational Biology, MRC Weatherall Institute of Molecular Medicine, University of Oxford, Oxford, OX3 9DS, United Kingdom; MRC Molecular Haematology Unit, MRC Weatherall Institute of Molecular Medicine, University of Oxford, Oxford, OX3 9DS, United Kingdom; MRC WIMM Centre for Computational Biology, MRC Weatherall Institute of Molecular Medicine, University of Oxford, Oxford, OX3 9DS, United Kingdom; MRC Molecular Haematology Unit, MRC Weatherall Institute of Molecular Medicine, University of Oxford, Oxford, OX3 9DS, United Kingdom; MRC WIMM Centre for Computational Biology, MRC Weatherall Institute of Molecular Medicine, University of Oxford, Oxford, OX3 9DS, United Kingdom; MRC Molecular Haematology Unit, MRC Weatherall Institute of Molecular Medicine, University of Oxford, Oxford, OX3 9DS, United Kingdom; MRC WIMM Centre for Computational Biology, MRC Weatherall Institute of Molecular Medicine, University of Oxford, Oxford, OX3 9DS, United Kingdom; MRC Molecular Haematology Unit, MRC Weatherall Institute of Molecular Medicine, University of Oxford, Oxford, OX3 9DS, United Kingdom; MRC WIMM Centre for Computational Biology, MRC Weatherall Institute of Molecular Medicine, University of Oxford, Oxford, OX3 9DS, United Kingdom

## Abstract

**Summary:**

Genome-wide association studies have revealed that many disease-associated genetic variants lie in non-coding regions of the genome. To prioritize these variants and clarify their functional roles, accurate classification of *cis*-regulatory elements is essential. Early approaches relied on characteristic histone marks, while more recent methods use Hidden Markov Models to segment the genome into chromatin states. However, these models often produce abstract states that require manual interpretation to assign regulatory function. REgulamentary is introduced as a rule-based framework for *de novo*, genome-wide annotation of *cis*-regulatory elements in a cell type-specific manner. Its behaviour is compared with count-based and segmentation-based approaches to highlight differences in classification strategy and the interpretability advantages of a rule-based design. Finally, its utility in the analysis of complex disease *loci* is demonstrated through application to published genetic association data to prioritize likely causal variants.

**Availability and implementation:**

REgulamentary is implemented in Python with a Snakemake-based workflow for reproducible analysis, integrating standard bioinformatics tools. The software is available at: https://github.com/Genome-Function-Initiative-Oxford/REgulamentary

## 1 Introduction

The non-coding genome is known to be populated with potentially millions of regulatory elements which act to control spatiotemporal gene expression. Two of the most important classes of regulatory elements are Enhancers and Promoters which have specific but overlapping roles. Promoters act to enable transcription initiation, they are typically short sequences, located close to the transcription start site (100 bp–1 kb). Active Promoters, that is those associated with actively transcribed genes, reside in regions of open chromatin, and display the characteristic tri-methylation of Histone H3 at Lysine 4 (H3K4me3). Promoter elements contain general transcription factor binding motifs and thus act as a platform on which a compendium of transcription factors can bind and associate with general transcription machinery in order to initiate transcription. Enhancer elements on the other hand, are located more distally from the target gene, at distances in excess of 1 Mb in some cases. Enhancer sequences are known to contain the cell type-specific transcription factor binding sites that encode the specificity of gene expression. When active, Enhancers exhibit the characteristic histone signatures: mono-methylation of Histone H3 at Lysine 4 (H3K4me1) and acetylation of the lysine residue at N-terminal position 27 of the histone H3 (H3K27ac), and recruit tissue-specific transcription factors in order to regulate cell type-specific gene expression (Larke *et al.* 2021, Downes and Hughes 2022).

The classical definitions of Enhancers and Promoters stated above are somewhat oversimplified. It is well known that there is a high degree of overlap between the histone modifications displayed by active Enhancers and Promoters. It is not possible to predict which target genes an Enhancer will interact with by genomic position alone, Enhancers often skip the most proximal genes and may interact with more than one gene within a given locus, thus metrics such as distance to the nearest transcription start site (TSS) can often be misleading. For many purposes it is of interest to determine the activity of *cis*-regulatory elements, for example, to know whether they are active in a given cell type. To directly measure the activity of a *cis*-regulatory element is challenging, therefore it is common practice to infer the activity of an element based on chromatin accessibility, associated histone marks, and transcription factor binding. However, given the degree of overlap in these classifiers across Enhancers and Promoters, this is not a simple task, and depending on the chosen method this can give varying results (Larke *et al.* 2021, Downes and Hughes 2022).

**Figure 1:**
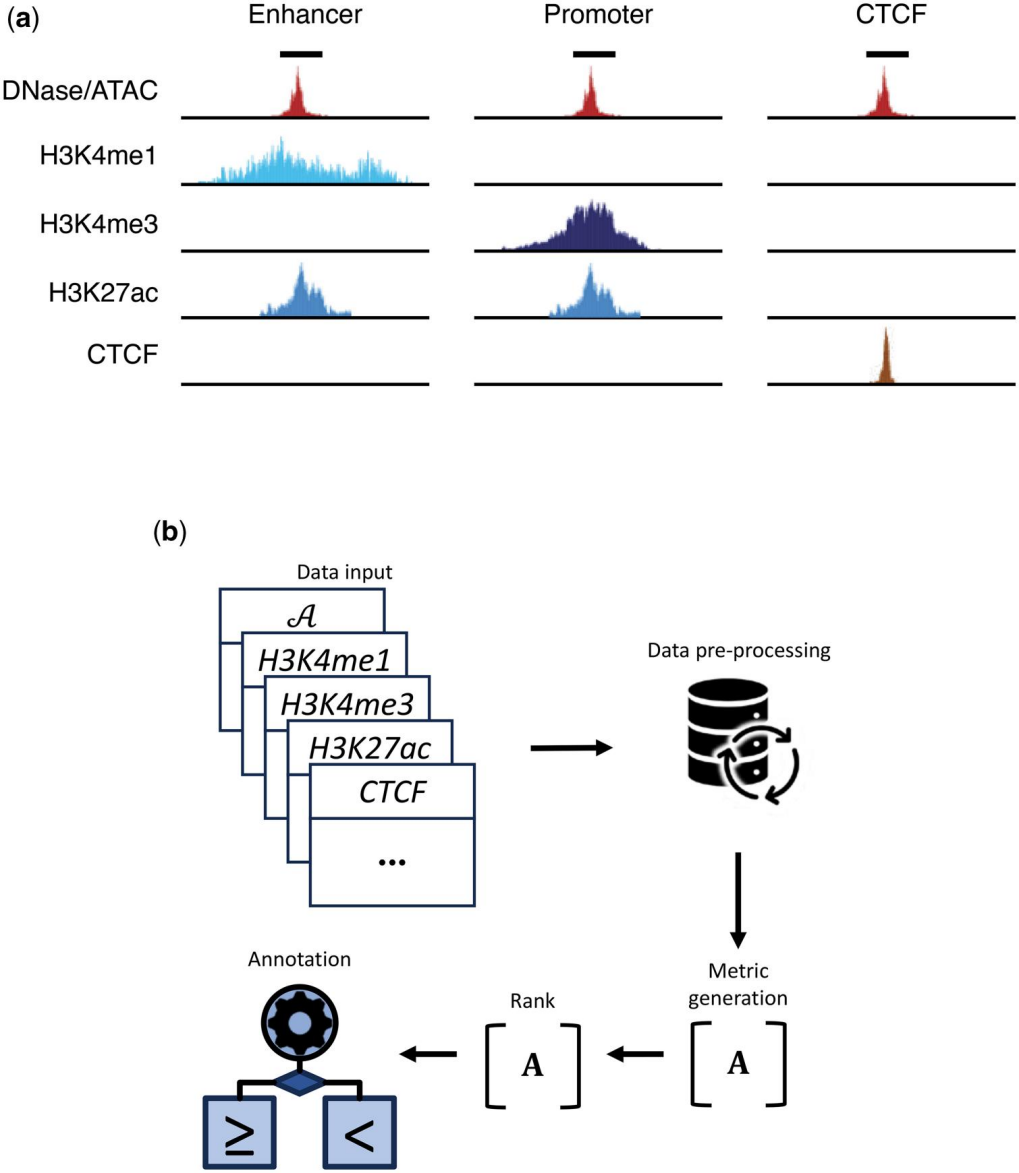
(a) Visualization of how regulatory elements are identified by using chromatin accessibility data (such as DNase, bulk-ATAC, or single-cell ATAC-seq data), H3K4me1, H3K4me3, H3K27ac histone modification marks, and ChIP CTCF-seq data. (b) Graphical overview of REgulamentary.

Active regulatory elements—visually shown in Fig. 1a—are located in regions of open chromatin and marked by specific features, which can be used to putatively identify them in the genome. H3K4me3 and H3K27ac are associated with active Promoters, whereas H3K4me1 and H3K27ac are found at active Enhancers (Larke *et al.* 2021, Oudelaar and Higgs 2021, Downes and Hughes 2022, Herrmann *et al.* 2022). Boundary elements are an additional class of *cis*-regulatory elements, which in contrast, are predominantly characterized by the binding of the CCCTC-binding factor (CTCF) (Kim *et al.* 2007, Oudelaar and Higgs 2021). Classification of regulatory elements within any given eukaryotic cell type, therefore, requires: (i) the positions of regions of open chromatin based on either DNase-seq (Thurman *et al.* 2012), ATAC-seq (Buenrostro *et al.* 2015a) or single-cell ATAC-seq (Buenrostro *et al.* 2015b), (ii) genome-wide data for the histone modifications H3K27ac, H3K4me1 and H3K4me3 based on ChIP-seq, and (iii) genome-wide data for CTCF based on ChIP-seq (Barski *et al.* 2007, Johnson *et al.* 2007). Further details on the input files required for REgulamentary can be found in the Section 2.

There have been a number of bioinformatic tools built for the task of *cis*-regulatory element classification, however, there is a lack of a substantial ground-truth dataset in which both Enhancer and Promoter elements have been experimentally validated. In addition, the identification of regulatory elements is data-driven, high-quality and cell type-specific input data is required in order to achieve an accurate classification. Unsupervised learning methods might be suited to this task; however, these methods are not able to assign regulatory elements. Instead, they group genomic regions by their similarities. For this reason, we opted for a more systematic approach. In addition to Hidden Markov Model (HMM)-based segmentations, several rule-based frameworks have been widely adopted (Ernst and Kellis 2012, Zacher *et al.* 2017). The ENCODE cCRE Registry uses accessibility anchors and rule-based criteria on DNase/ATAC, H3K4me3, H3K27ac, and CTCF to assign promoter-like, enhancer-like, and related classes, with per-biosample activity calls. StateHub/StatePaintR provides a decision-matrix framework to assign chromatin states from epigenomic peaks in a transparent and reproducible manner (Coetzee *et al.* 2020). Unsupervised HMM-based methods (e.g. ChromHMM, GenoSTAN) are highly flexible in the epigenomic marks they ingest and can discover data-driven subclasses of promoters and enhancers while providing genome-wide state maps, including weak or poised chromatin states. ChromHMM is widely adopted in large consortia and provides robust genome-wide context, whereas GenoSTAN is a count-based HMM with strong published benchmarks for promoter and enhancer identification. In contrast, rule-based approaches offer direct interpretability but are typically anchored to specific genomic signals. Our goal is to provide an interpretable, anchor-centric framework that complements these existing strategies. For these reasons, in this work, we propose REgulamentary, a rule-based, anchor-centric method that directly labels accessible *loci* as Promoter, Enhancer, CTCF, or hybrid classes using a minimal assay set (H3K4me3, H3K27ac, H3K4me1, CTCF, and ATAC/DNase). It is designed to complement genome-wide segmentations (e.g. ChromHMM, GenoSTAN) and curated resources such as the ENCODE cCRE Registry (Moore *et al.* 2020), by providing portable, dataset-specific labels without requiring a state-mapping step. REgulamentary takes into account the characteristics of each element, as they would be manually assigned by an expert biologist, in an automated and genome-wide manner.

## 2 Methods

REgulamentary takes the following input data types: (i) chromatin accessibility data (this can be either ATAC, scATAC, or DNase), (ii) three histone mark ChIP-seq data sets (H3K4me1, H3K4me3, and H3K27ac), and (iii) ChIP-seq for the CTCF boundary element. For data types (ii) and (iii), aligned sequences (BAM format), coverage (bigWig format), and peak files (bed format) are required, whereas for chromatin accessibility only the peak bed file is necessary. Table 1 shows the data that has to be provided to REgulamentary, whilst Fig. 1b illustrates the graphical overview of the proposed method. REgulamentary has been implemented using Python (v3.9.10) and we provided all packages/libraries with their relative versions in Table 1, available as supplementary data at *Bioinformatics Advances* online.

**Table 1:** The data formats required as input to REgulamentary.^a^

	Chromatin accessibility (A)	H3K4me1	H3K4me3	H3K27ac	CTCF
BAM		X	X	X	X
bigWig		X	X	X	X
BED	X	X	X	X	X

a Boxes marked with X are required data types. BAM, bigWig, and bed files for each target must originate from each other, e.g. the CTCF BED must be a bed file containing peaks called from the provided CTCF bigWig file, where peaks are naturally variable in size.

### 2.1 Step 1: data pre-processing

For pre-processing and meta-plot visualization, REgulamentary first defines A as the set of chromatin accessibility peaks (ATAC or DNase). It then concatenates, sorts by coverage in ascending order, and identifies the unique peaks between A and *CTCF* peak files to create the list of union peaks ($\underline{r}$). From $\underline{r}$, peaks intersecting with blacklist regions are removed. Using the multi-coverage function within bedtools (Quinlan and Hall 2010), a $C_{r}$ coverage matrix is calculated for each $r\in \underline{r}$ for *H3K4me1* and *H3K4me3* histone-mark ChIP-seq data. Within $C_{r}$, each $r\in \underline{r}$ is then ranked in descending order based on the difference between the coverage values of *H3K4me3* and *H3K4me1*.

### 2.2 Step 2: metric generation

Let *auc* (area under the curve—read count for provided regions) (DeLong *et al.* 1988), be a function defined as follows: $\mathrm{auc}:\underline{r}\to N_{\geq 0}$, where auc(r)=read coverage of r. After data pre-processing, REgulamentary intersects $\underline{r}$, the regions of interest, with the three histone mark ChIP-seq and the CTCF boundary element and uses *auc* to calculate the normalized—per genome-wide sequencing depth—read counts, creating a R×T matrix $A_{r}^{t}$ [Equation (1)], where $R=|\underline{r}|$ and T=4, which represents the four tracks: $\underline{t}=[\mathrm{H3K4me1,H3K4me3,H3K27ac},\mathrm{CTCF}\;]$. $A_{r}^{t}$ is defined as:


(1) $$\forall \;r\in \underline{r}\;\wedge \;\forall \;t\in \underline{t}:A_{r}^{t}=1e6\cdot \frac{\mathrm{auc}(r\cap b^{t})}{\mathrm{auc}{\left(\mathrm{genome}\right)}^{t}},$$

where $b^{t}$ indicates the regions of interest for $t\in \underline{t}$.

### 2.3 Step 3: Rank

Given $t_{1}=\mathrm{H3K4me1}$, $t_{2}=\mathrm{H3K4me3}$, $t_{3}=\mathrm{H3K27ac}$, and $t_{4}=\mathrm{CTCF}$, let *rank* be a function defined as follows: $\mathrm{rank}:R_{\geq 0}^{4}\to [1,2,3,4]$. For each $r\in \underline{r}$, $A_{r}:=[A_{r}^{t_{1}},A_{r}^{t_{2}},A_{r}^{t_{3}},A_{r}^{t_{4}}]$ is ranked in descending order, accordingly to $A_{r}$’s values, creating a R×T matrix $R_{r}^{t}$ where [Equation (2)]:


(2) $$\forall \;r\in \underline{r}:R_{r}:=[R_{r}^{t_{1}},R_{r}^{t_{2}},R_{r}^{t_{3}},R_{r}^{t_{4}}]=\mathrm{rank}(A_{r},\;\text{order}=\text{descending}).$$

### 2.4 Step 4: annotation

Finally, REgulamentary systematically assigns to each $r\in \underline{r}$ a regulatory element (RE) label (namely Enhancer, Promoter, CTCF, Enhancer/CTCF, and Promoter/CTCF) in two phases. The first phase assigns the main RE: Enhancer, Promoter, and CTCF, by applying the following rule (3):


(3) $$\begin{array}{c} \forall \;r\in \underline{r}:\\ {\text{RE}}_{r}=\{\begin{array}{cc} \text{Enhancer} & \text{if}\;\begin{array}{c} (R_{r}^{t_{1}}=1\wedge R_{r}^{t_{2}}=2)\;\vee \\ (R_{r}^{t_{1}}=1\wedge R_{r}^{t_{3}}=2)\;\vee \\ (R_{r}^{t_{1}}=2\wedge R_{r}^{t_{3}}=1)\;\vee \\ (R_{r}^{t_{1}}=1\wedge A_{r}^{t_{2}}=0\;\wedge \\ \;A_{r}^{t_{3}}=0\wedge A_{r}^{t_{4}}=0) \end{array}\\ \text{Promoter} & \text{if}\;\begin{array}{c} (R_{r}^{t_{2}}=1\wedge R_{r}^{t_{1}}=2)\;\vee \\ (R_{r}^{t_{2}}=1\wedge R_{r}^{t_{3}}=2)\;\vee \\ (R_{r}^{t_{2}}=2\wedge R_{r}^{t_{3}}=1)\;\vee \\ (R_{r}^{t_{2}}=1\wedge A_{r}^{t_{1}}=0\;\wedge \\ \;A_{r}^{t_{3}}=0\wedge A_{r}^{t_{4}}=0) \end{array}\\ \text{CTCF} & \text{if}\;\begin{array}{c} (R_{r}^{t_{4}}=1\wedge R_{r}^{t_{3}}=2)\;\vee \\ (R_{r}^{t_{4}}=2\wedge R_{r}^{t_{3}}=1)\;\vee \\ (R_{r}^{t_{4}}=1\wedge A_{r}^{t_{1}}=0\;\wedge \\ \;A_{r}^{t_{2}}=0\wedge A_{r}^{t_{3}}=0) \end{array}\\ \text{Not}\;\text{assigned} & \text{otherwise} \end{array} \end{array}$$

whilst the second phase tries to discriminate Enhancer or Promoter regions which are co-accessible with CTCF sites (Enhancer/CTCF and Promoter/CTCF), by applying (4):


(4) $$\begin{array}{c} \forall \;r\in \underline{r}:\\ {\text{RE}}_{r}=\{\begin{array}{cc} \text{Enhancer}/\text{CTCF} & \text{if}\;\begin{array}{c} (R_{r}^{t_{4}}=1\wedge R_{r}^{t_{1}}=2\;\wedge \\ \;A_{r}^{t_{1}}\neq 0\wedge A_{r}^{t_{4}}\neq 0)\;\vee \\ (R_{r}^{t_{4}}=2\wedge R_{r}^{t_{1}}=1\;\wedge \\ \;A_{r}^{t_{1}}\neq 0\wedge A_{r}^{t_{4}}\neq 0)\;\vee \\ (A_{r}^{t_{1}}\neq 0\wedge A_{r}^{t_{2}}=0\;\wedge \\ \;A_{r}^{t_{3}}\neq 0\wedge A_{r}^{t_{4}}\neq 0) \end{array}\\ \text{Promoter}/\text{CTCF} & \text{if}\;\begin{array}{c} (R_{r}^{t_{4}}=1\wedge R_{r}^{t_{2}}=2\;\wedge \\ \;A_{r}^{t_{2}}\neq 0\wedge A_{r}^{t_{4}}\neq 0)\;\vee \\ (R_{r}^{t_{4}}=2\wedge R_{r}^{t_{2}}=1\;\wedge \\ \;A_{r}^{t_{2}}\neq 0\wedge A_{r}^{t_{4}}\neq 0)\;\vee \\ (A_{r}^{t_{1}}=0\wedge A_{r}^{t_{2}}\neq 0\;\wedge \\ \;A_{r}^{t_{3}}\neq 0\wedge A_{r}^{t_{4}}\neq 0) \end{array} \end{array} \end{array}$$

To summarize, REgulamentary assigns regulatory element labels only at genomic *loci* that overlap either (i) peaks of chromatin accessibility (ATAC/DNase) or (ii) CTCF-binding peaks. These anchors define the set of candidate regulatory elements for which histone-mark and CTCF signals are quantified. Regions lacking accessibility or CTCF signal are not annotated, as the method is designed to classify elements with evidence of regulatory potential rather than to segment the entire genome. Enhancer annotations are further subdivided into *active* and *inactive* subclasses based on the presence or absence of H3K27ac signal. Specifically, an enhancer is considered *active* when its normalized H3K27ac signal exceeds the threshold described; otherwise, it is assigned to the *inactive* class. These subclass definitions reflect established chromatin biology, in which H3K27ac marks active enhancers, whereas H3K4me1, in the absence of H3K27ac, typically denotes inactive elements.

## 3 Results

From ENCODE we downloaded publicly available FASTQ files for: DNase-seq (*n = *14), ChIP-seq of the histone modifications H3K27ac (*n = *2), H3K4me1 (*n = *3), and H3K4me3 (*n = *7) and ChIP-seq of CTCF (*n = *7) in human umbilical vein endothelial cells (HUVECs) (de Souza 2012) (Table 2, available as supplementary data at *Bioinformatics Advances* online, for ENCODE accession sample ids). The FASTQ files were first processed using the complete ATAC-seq and ChIP-seq upstream pipeline (CATCH-UP) (Riva *et al.* 2023), a portable automated pipeline for analysing bulk ATAC-seq and ChIP-seq data. In addition to BAM files, CATCH-UP outputs bigWig files for visualization and BED files containing the peak calls for each sample, which can be readily used as input for REgulamentary.

**Table 2:** Manual assessment of GenoSTAN states, based on (log) scaled read counts (Fig. 3a), according to the REgulamentary naming assignment.

State	Annotation
1	Not assigned
2	Promoter
3	CTCF
4	Enhancer
5	Promoter/CTCF
6	Promoter

### 3.1 REgulamentary result

As stated in Section 2.1, all regions of interest (total of 33 792 sites) intersect chromatin accessibility regions from DNase-seq, and have variable CTCF coverage.

**Figure 2:**
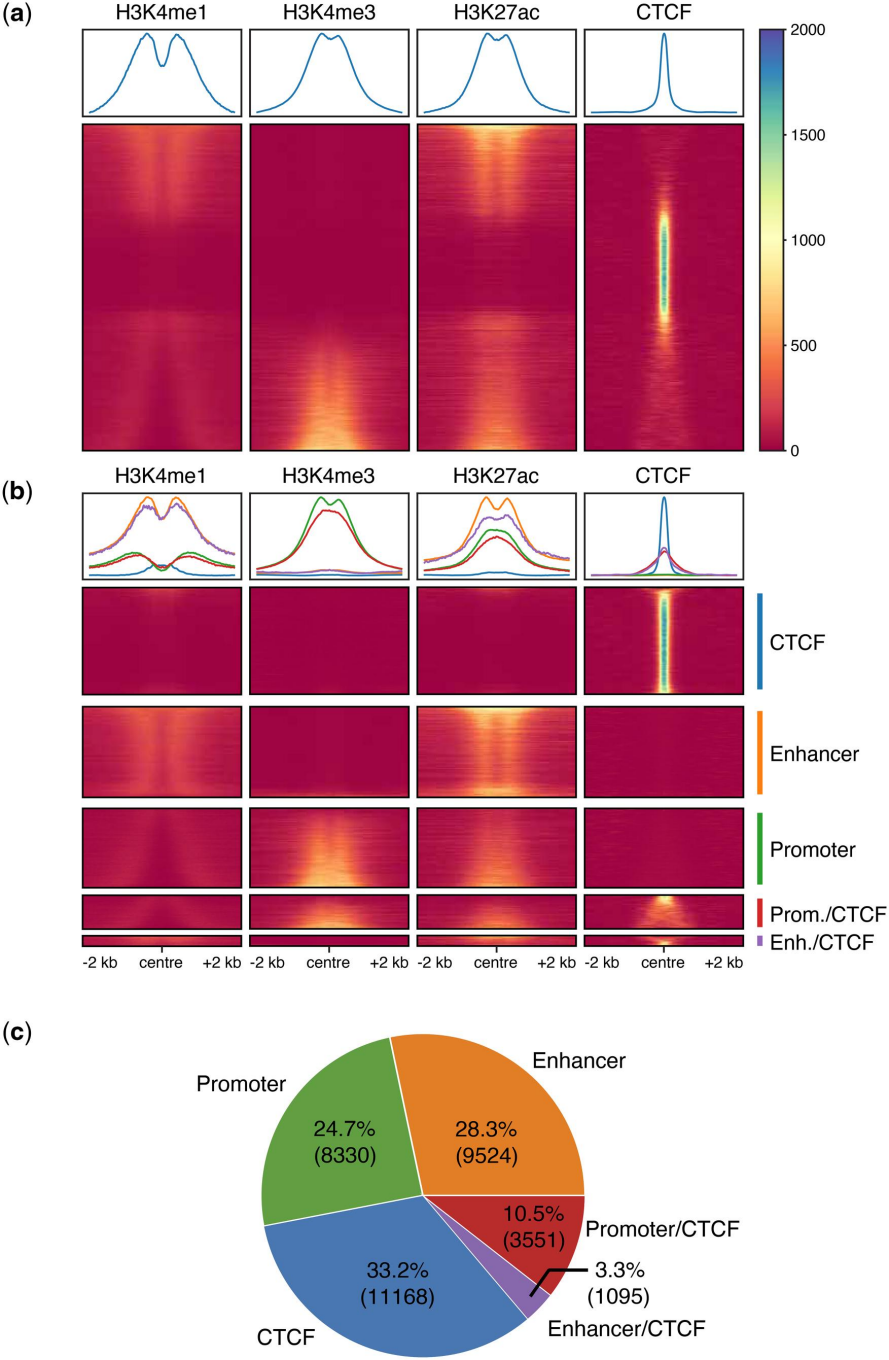
In (a) is shown the meta-plot of the coverage ±2kb from the centre of the sorted regions of interest in HUVECs (as explained in Section 2.1), whilst (b) shows the same meta-plot grouped instead by regulatory element assigned by REgulamentary. (c) The piechart displays the distribution of regulatory elements in HUVEC regions of interest.

Figure 2a shows the 33 792 peak regions of interest, derived from the processed HUVEC DNase-seq data. These regions of interest were then assigned to specific classes of regulatory elements using REgulamentary, and are grouped together in Fig. 2b. Out of the 33 792 regions of interest, 33 669 (>99.996%) were assigned as regulatory elements, demonstrating the thoroughness with which REgulamentary assigns a regulatory element to each region of interest. The most commonly identified regulatory elements were CTCF site (33.3%), followed by Enhancers (28.3%) and Promoters (24.7%), as shown in Fig. 2c. A small fraction of the regions showed characteristics of either a Promoter or an Enhancer that overlapped with CTCF binding (10.5% and 3.3%, respectively). For completeness, enhancer calls are further subdivided into *active* and *inactive* subclasses based on their H3K27ac signal (Creyghton *et al.* 2010), using the thresholds defined in the Section 2.

### 3.2 Benchmarking against GenoSTAN

**Figure 3:**
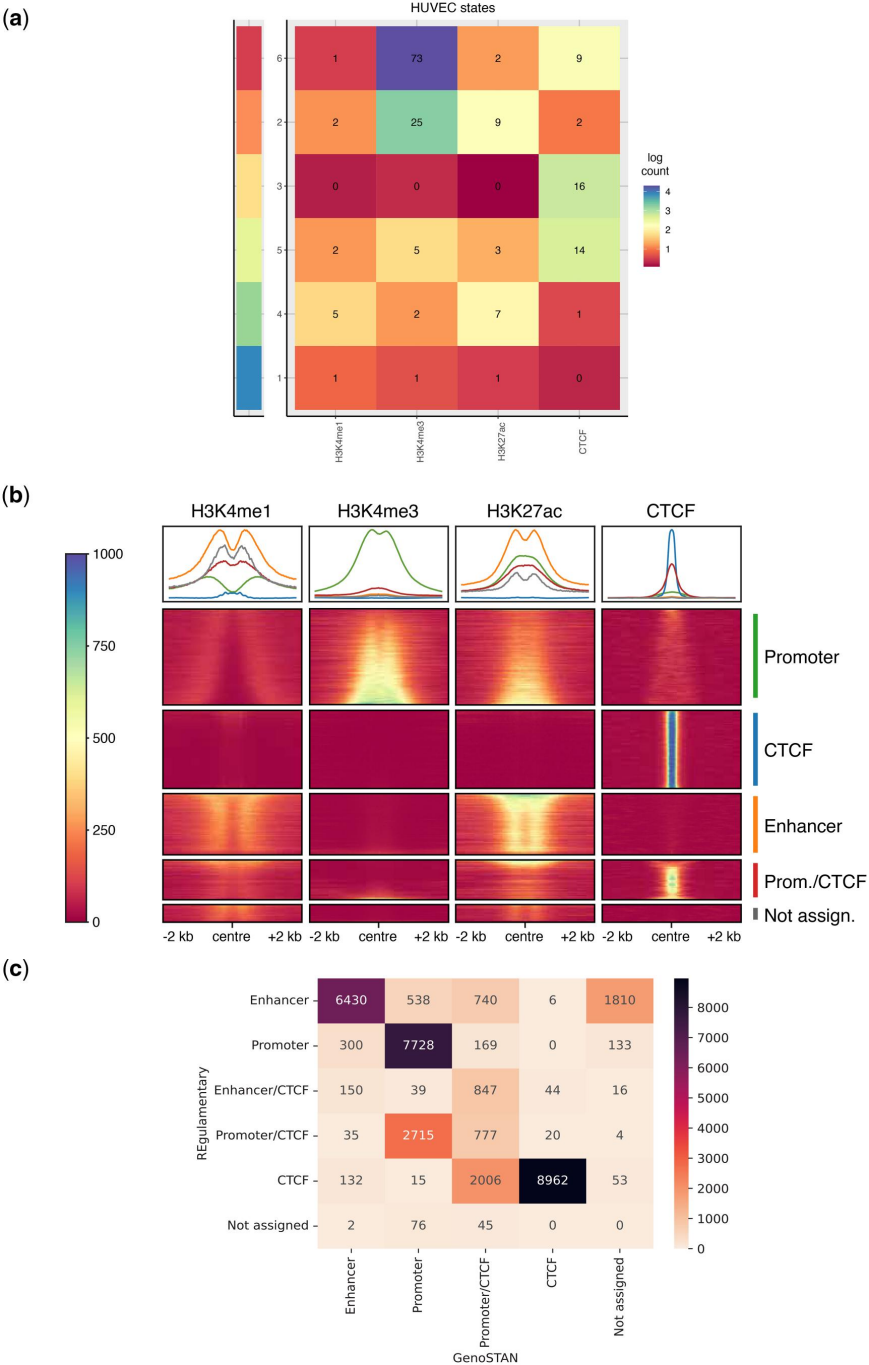
(a) The heatmap shows the distribution of log normalized count for each GenoSTAN state across H3K4me1, H3K4me3, H3K27ac, and CTCF. (b) The meta-plot displays the coverage ±2kb from the centre of the sorted (as explained in Section 2.1) regions of interest in HUVECs grouped by the manual assignment (Table 2) of the regulatory elements to the GenoSTAN states. (c) The confusion matrix displays the comparison in absolute numbers of the regulatory elements between REgulamentary (*y*-axis) and GenoSTAN (*x*-axis) outputs, respectively.

For benchmarking, we selected GenoSTAN as a representative count-based HMM framework due to its flexible multivariate emission model and strong published performance in promoter and enhancer identification across ENCODE and Roadmap datasets. We also acknowledge that ChromHMM is widely adopted for large-scale, cross–cell type chromatin state annotation; our goal is therefore not to imply exclusivity, but to position REgulamentary alongside commonly used HMM approaches. GenoSTAN is implemented within the R/Bioconductor STAN package (Zacher *et al.* 2014) and learns chromatin states using Hidden Markov Models with flexible multivariate count distributions, providing a well-established baseline for comparison. For comparison purposes, we initialise and fit (bidirectionally) GenoSTAN with six states and use a Gaussian emission distribution for the model. For each region of interest, we calculate the most likely state path using Viterbi. We emphasize that GenoSTAN is capable of learning a substantially larger number of latent chromatin states; however, here we selected six states solely to enable a like-for-like comparison with the six regulatory classes produced by REgulamentary (Promoter, Enhancer, CTCF, Promoter/CTCF, Enhancer/CTCF, and Not assigned). Using a matched number of output classes avoids introducing an additional, subjective state-to-label mapping step for GenoSTAN and allows the comparison to focus on differences in classification behaviour rather than differences in model granularity. This choice therefore reflects a benchmarking design consideration rather than an intrinsic limitation of GenoSTAN. After the assignment of the chromatin states, we used a heatmap plot (Fig. 3a), showing the normalized read counts per state for H3K4me1, H3K4me3, H3K27ac, and CTCF to assess the regulatory elements, which have been manually assigned based on visual assessment (Table 2) to each GenoSTAN state, according to the normalized read counts and according to the naming automatically given by REgulamentary. Differences between REgulamentary and GenoSTAN do not necessarily indicate misclassification; in many cases, they reflect alternative but biologically plausible interpretations of *loci* with overlapping chromatin features.

As a first visual result, we re-plot the meta-plot for comparison purposes, this time grouping regions of interest based on GenoSTAN state annotation in Fig. 3b, where it is possible to see that some regions were miss-classified by using the GenoSTAN approach. For example, the Promoter/CTCF group contains a significant subset of regions with a high coverage of H3K4me1, a mark characteristic of Enhancer classes, suggestive of a miss-classification.

**Figure 4:**
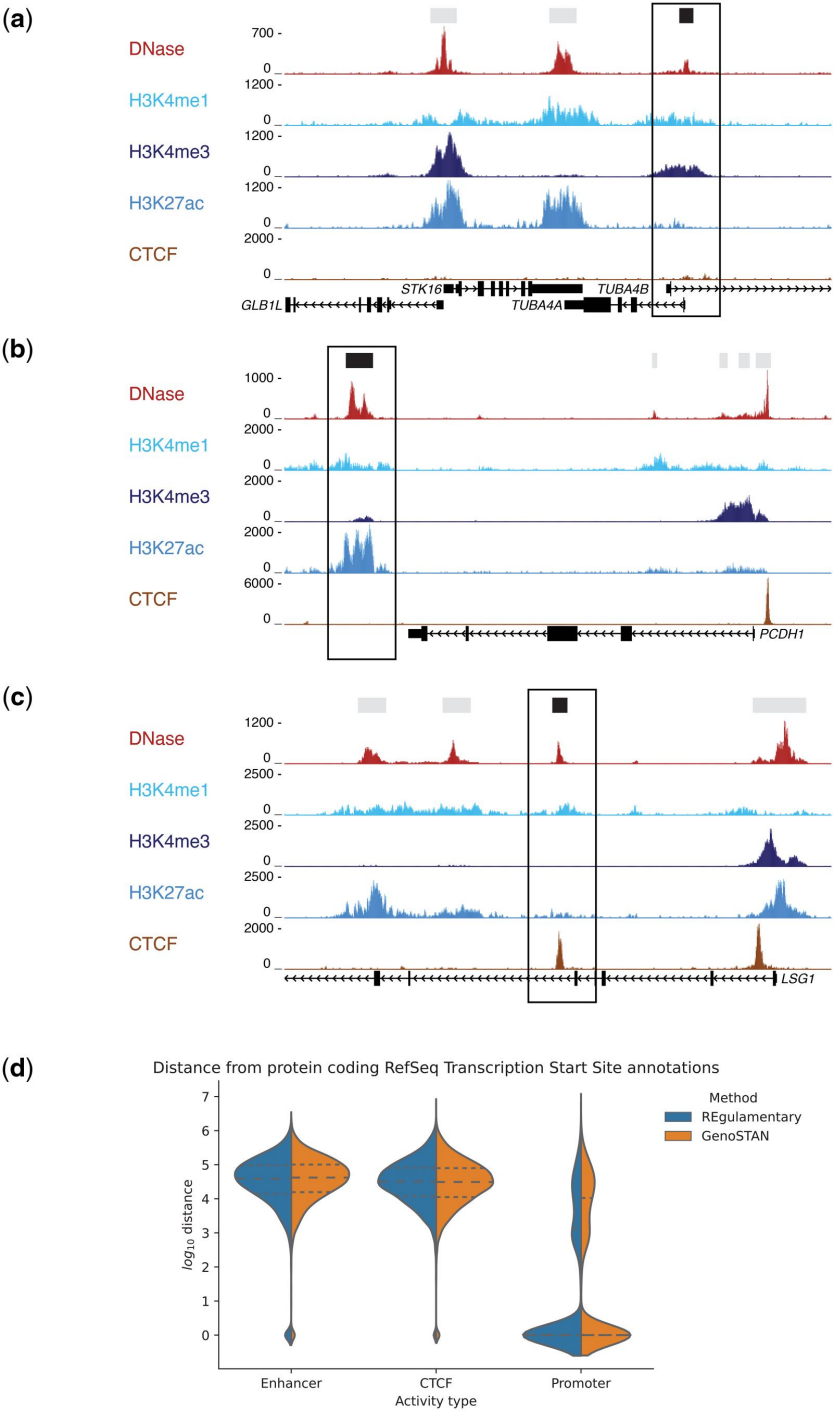
Highlighted, in (a) is shown a correctly assigned Promoter region by REgulamentary but misassigned as Enhancer by GenoSTAN. Likewise for (b), a correct Enhancer region for REgulamentary and misassigned by GenoSTAN as Promoter. Lastly, in (c), a properly annotated CTCF region by REgulamentary and misassigned Enhancer by GenoSTAN. (d) Violin plot of *log*_10_ distance of regulatory elements to RefSeq defined TSS. We observe that GenoSTAN has more defined Promoter elements far from RefSeq TSSs.

In order to compare REgulamentary against GenoSTAN, it is important to understand how closely the classifications of each of the 33 792 regions of interest agree (Fig. 3c). First, we show that for 2715 regions the output of the two tools closely align: REgulamentary identifies as Promoter/CTCF and GenoSTAN as Promoter. However, approximately a third of the total regions of interest (9895 out of 33 792) were classified differently by REgulamentary and GenoSTAN. Of interest are the 300 regions that REgulamentary identifies as Promoter and GenoSTAN identifies as Enhancer, representing a high degree of mismatch between the two methods. This mismatch is also observed in the 538 Enhancer-Promoter (REgulamentary-GenoSTAN, respectively), and the 132 CTCF-Enhancer (REgulamentary-GenoSTAN, respectively). In these cases, we randomly selected an example of each of these classes to manually investigate these discrepancies. For example, the Promoter of TUBA4A, a gene which encodes a key component of cytoskeletal microtubules, is correctly assigned by REgulamentary, but incorrectly assigned as an Enhancer by GenoSTAN (Fig. 4a). This Promoter exhibits both H3K4me1 and H3K4me3 signals, which may explain the discrepancy. However, REgulamentary is able to correctly identify this feature due to the correct ranking of the relative signal strength of the two chromatin marks. Similarly, an Enhancer located downstream of the vascular endothelial surface protein PCDH1 gene which exhibits high levels of H3K4me1 and low levels of H3K4me3 is correctly identified by REgulamentary, but misidentified as a Promoter by GenoSTAN (Fig. 4b). Finally, a CTCF site located in an intron of the LSG1 gene, which also exhibits some diffuse nearby H3K4me1 signal, again, is correctly identified by REgulamentary but misassigned as an Enhancer by GenoSTAN (Fig. 4c).

### 3.3 Intersection with reference sequence gene annotation

We define transcription start sites (TSSs) using Reference Sequence (Goldfarb *et al*. 2025) database annotation (version GCF_000001405.40) (Larke *et al.* 2021, RefSeq). Genes are filtered to include genes labelled as protein-coding, microRNA (miRNA), or long non-coding RNAs (lncRNA). TSSs are defined using the gene’s start if forward-strand or the gene’s end if reverse-strand. For each regulatory element, we define an annotation called $\mathrm{Log}.\mathrm{Distance}.\mathrm{TSS}\text{:}=log_{10}(d+1)$ where *d* is the distance to the closest transcription start site. If the regulatory element overlaps the distance is defined as 0 (so Log.Distance.TSS=0). Figure 4d shows how most Enhancer or CTCF (pure CTCF elements) regulatory elements are far from the TSSs whether in both methods (REgulamentary and GenoSTAN). However, we see that for both methods there is a subset of defined Promoter elements which are distal from defined TSSs, suggesting they are actually Enhancer elements. As seen in Fig. 4d, we observe that GenoSTAN misclassified Enhancers as Promoters more than REgulamentary. As a support, in Fig. 1, available as supplementary data at *Bioinformatics Advances* online, we compared REgulamentary and GenoSTAN annotated regulatory elements to the $log_{10}$ read-counts from cap analysis of gene expression (CAGE), a standard method for measuring TSS—see Table 3, available as supplementary data at *Bioinformatics Advances* online—(Shiraki *et al.* 2003), H3K4me1 ChIP, H3K4me3 ChIP, and CTCF CHiP, plotting the count distributions in a similar manner to Fig. 4d. Future benchmarking could also incorporate experimentally supported regulatory element sets, such as FANTOM5 CAGE-defined promoters (Nobusada *et al.* 2025), ENCODE cCREs, or STARR-seq enhancer screens (Arnold *et al.* 2013), providing orthogonal functional evidence to complement the comparisons shown here.

### 3.4 Intersection with GWAS

**Figure 5:**
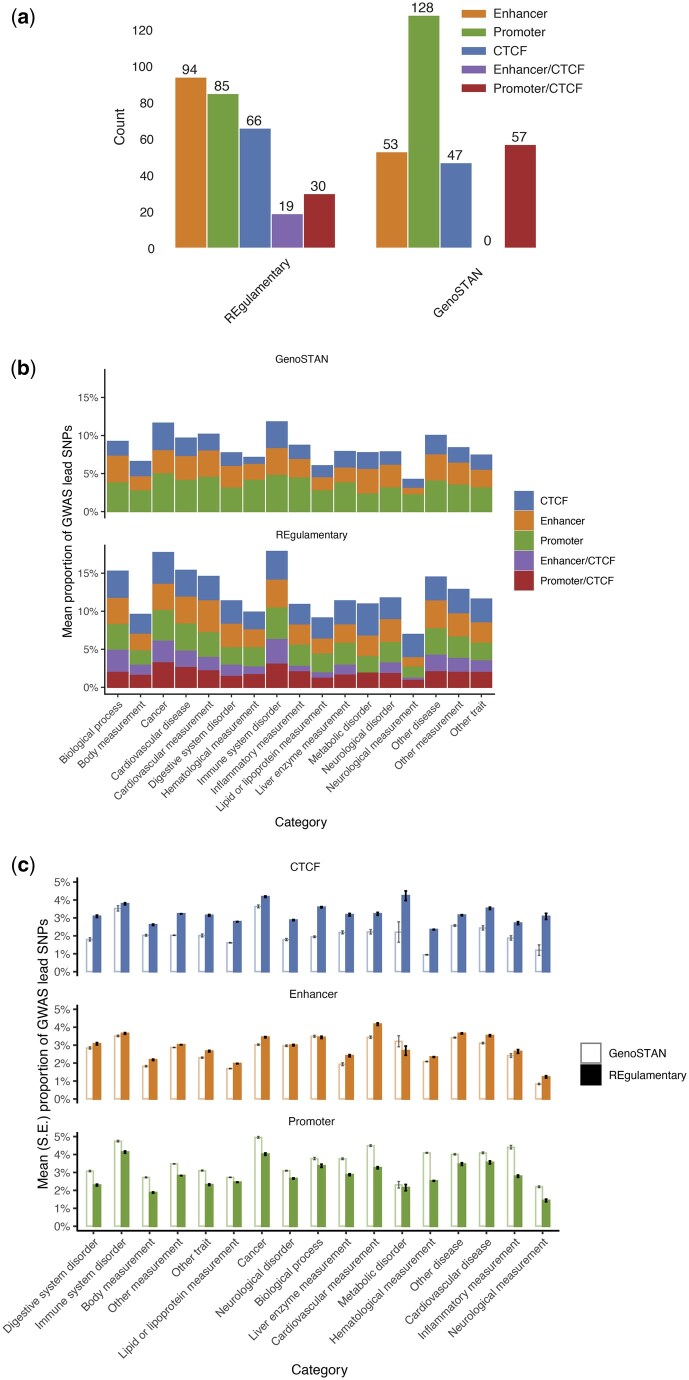
(a) The barplot shows the number of stroke GWAS genetics intersection with REgulamentary and GenoSTAN annotated regulatory elements, respectively. (b) Proportion of GWAS lead variants intersecting GenoSTAN (top) and REgulamentary (bottom) annotated genomic elements. The mean proportion of annotated variants was calculated across all 4949 GWASs with at least 10 lead variants reported in GWAS Catalogue version 1.0.2 (downloaded on 22 August 2023). GWASs were annotated to categories based on EFO mappings. (c) Proportion of GWAS lead variants intersecting CTCF, Enhancer, and Promoter elements annotated by REgulamentary (filled) or GenoSTAN (unfilled) across disease categories. The mean and standard error (SE) of the proportion of annotated variants was calculated across all 4949 GWASs with at least 10 lead variants reported in GWAS Catalogue version 1.0.2 (downloaded on 22 August, 2023). GWASs were annotated to categories based on Experimental Factor Ontology (EFO) mappings.

Prioritization of the common non-coding genetic variants found in GWAS requires the accurate identification of cell type-specific regulatory elements. This is a critical step because the potential mechanism of causality of the variant will be very different depending on the type of element affected, as will the experiments required to test for causality of the variant. To demonstrate the utility of REgulamentary, we selected and downloaded stroke-related lead SNPs from the GWAS Catalogue (Sollis *et al.* 2023), version 1.0.2, downloaded on 22 August 2023 (see Table 4, available as supplementary data at *Bioinformatics Advances* online, for a comprehensive list), and imputed proxy SNPs, $R^{2}$ filtered ($R^{2}\geq 0.8$), by using TopLD (Huang *et al.* 2022) on the European population. Stroke is a leading cause of morbidity and mortality in the world, with a significant genetic component, and represents a large unmet clinical need. The underlying pathological mechanisms are thought to involve endothelial cells, therefore we predict that a proportion of the genetic associations will be active in HUVECs (Andjelkovic *et al.* 2019, Mishra *et al.* 2022). In total, 17 571 stroke-associated imputed SNPs were intersected with the REgulamentary defined HUVEC regulatory elements. 94 SNPs were identified in Enhancers, along with 85 in Promoters, and 65 in CTCF elements. A further 19 and 30 were identified in Enhancer/CTCF and Promoter/CTCF elements (Fig. 5a).

We then extended this analysis to all 4949 studies across 17 trait and disease categories in the GWAS Catalogue (Sollis *et al.* 2023) with at least ten lead variants. Across all categories, a higher proportion of lead variants intersected with annotated REgulamentary elements than GenoSTAN elements (Fig. 5b). Moreover, a higher proportion of GWAS variants intersected annotated GenoSTAN rather than annotated REgulamentary Promoters across all 17 categories; but this trend was reversed to favour annotated REgulamentary over GenoSTAN Enhancers for 15/17 categories (Fig. 5c). These biases were especially apparent among GWASs of 173 cardiovascular and haematological measurements, as there was only one study for which more lead variants intersected REgulamentary over GenoSTAN-annotated Promoters; and only 15/173 studies for which more lead variants intersected GenoSTAN over REgulamentary-annotated Enhancers (Fig. 2, available as supplementary data at *Bioinformatics Advances* online).

The assignment of the elements containing these SNPs, differed significantly when the same analysis was run using GenoSTAN annotation of HUVECs, demonstrating the importance of an accurate annotation of cell type-specific regulatory elements. An accurate prioritization of disease-associated genetic variants may expedite the understanding of complex diseases and the search for novel therapies.

### 3.5 REgulamentary on 12 cell types

After showing detailed results and analyses in HUVEC, we collected open chromatin (DNase), H3K4me1, H3K4me3, H3K27ac, and CTCF data for 12 additional ENCODE cell types, namely: Astrocyte, B-cell, Cardiac-muscle-cell, CD4-positive_alpha-beta-t-cell, CD14-positive-monocyte, Fibroblast-of-dermis, Fibroblast-of-lung, Keratinocyte, Mammary-epithelial-cell, Natural-killer-cell, Osteoblast, and Skeletal-muscle-myoblast. We used CATCH-UP to process all FASTQs (Table 5, available as supplementary data at *Bioinformatics Advances* online), obtaining BAM and bigWig files. We then applied REgulamentary and GenoSTAN using the same parameters described in Section 3.2. Benchmarking against GenoSTAN. From GenoSTAN, we obtained the log-transformed median read coverage for each cell type (Fig. 3, available as supplementary data at *Bioinformatics Advances* online), which we used to annotate states manually (Table 6, available as supplementary data at *Bioinformatics Advances* online). Full comparisons for all 12 cell types are available as interactive Multi-Dimensional Viewer (MDV) projects (Weeratunga *et al.* 2023), and a summary of these resources is provided in Table 7, available as supplementary data at *Bioinformatics Advances* online. Across all cell types, REgulamentary produced annotation patterns consistent with those observed in HUVEC.

### 3.6 Interactive visualization of REgulamentary

**Figure 6:**
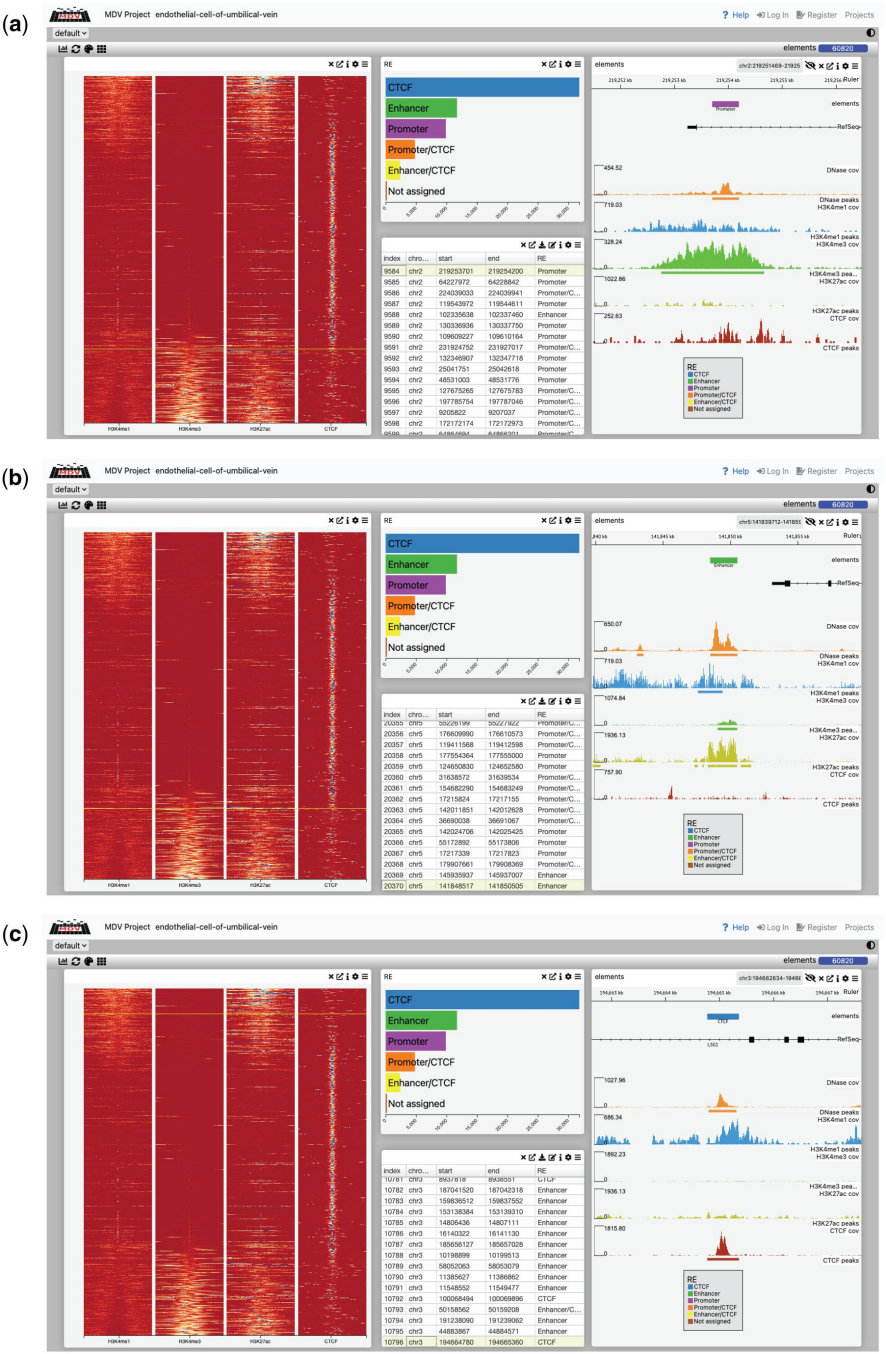
MDV genomic visualization showing heatmap analysis and corresponding data tracks across three different conditions (a–c). Each panel consists of three main components: (i) left: a metaplot displayed as a heatmap showing signal distribution patterns; (ii) centre: a detailed table listing genomic coordinates and annotations of regulatory elements, with a bar plot at the top showing their distribution across different categories; and (iii) right: coverage plots showing the distribution of genomic features across different elements. This visualization integrates multiple data representations to provide a comprehensive view of genomic features and their distribution across different experimental conditions in MDV.

To facilitate the usability and interpretation of REgulamentary results, they can be loaded into MDV with a single Python command. The results can be viewed locally or uploaded to a website for sharing. Potential regulatory elements can be sorted, filtered, and the underlying tracks for a particular region can be inspected in the built-in browser. An interactive meta-plot showing all the elements can be dynamically ordered, grouped, and have its colour settings adjusted. A combination of filtering and panning/zooming enables users to focus on fine details. Selecting a region on the metaplot displays information about that location and shows it in a genome browser. Conversely, selecting a region in a table, chart, or browser will highlight that region in the metaplot (Fig. 6a–c) show the potential of MDV, highlighting a Promoter region in a, an Enhancer region in b, and a CTCF region in c, with all three subfigures aligned with Fig. 4a–c. Projects for all 12 cell types mentioned in Section 3.5. REgulamentary on 12 cell types are available for inspection. Instructions on how to create a project and view the 12 cell types are available on the GitHub page.

## 4 Conclusion

In this work, we presented REgulamentary, a standalone, rule-based bioinformatic tool for the thorough annotation of *cis*-regulatory elements for chromatin-accessible or CTCF-binding regions of interest. We showed that the proposed tool is able to classify regions of interest accurately (Fig. 2b) and differs from GenoSTAN in ways that provide a more interpretable and biologically consistent classification (Fig. 3c), with illustrative examples shown in Fig. 4a–c. Compared with cCREs, which are a curated, cross-biosample registry generated by ENCODE’s centralized pipeline, REgulamentary is a user-run procedure for labelling peaks in new datasets. Compared with StatePaintR, which uses a flexible rule matrix to assign segment-level states across varied mark sets, REgulamentary uses fixed, quantitative rules at peak anchors and outputs direct promoter/enhancer/CTCF labels, including explicit/CTCF hybrids. We note that no single computational method should be regarded as a universal ‘gold standard’. The most appropriate reference depends on the analytic objective: for genome-wide context and large-scale, cross-cell type annotation, widely adopted tools such as ChromHMM [including the full-stack universal annotation (Vu and Ernst 2022)] remain the most practical foundation. For studies focused on per-cell type promoter/enhancer identification with count-based signal modelling, GenoSTAN retains appeal due to its strong published benchmarks (Zacher *et al.* 2017). Thus, REgulamentary is intended to complement, rather than replace, existing frameworks, offering a transparent anchor-centric classification when high-confidence regulatory elements are desired. Whilst we have shown that our tool performs favourably in comparison with established HMM-based approaches, we would like to highlight three key areas which will form the focus of future developments of this tool. Because REgulamentary operates at accessibility/CTCF anchors and uses a fixed five-mark rule set, it does not label histone-only sites (e.g. H3K4me1-only inactive enhancers) and does not discover additional sub-states beyond the defined macro-classes. For users requiring genome-wide coverage of weak or poised states, or highly flexible mark configurations, we recommend pairing REgulamentary with an HMM-based segmentation.

REgulamentary currently relies on a minimal set of widely available marks (H3K4me1, H3K4me3, H3K27ac, and CTCF) together with chromatin accessibility. While this design promotes portability across datasets, additional histone modifications, such as H3K27me3 (Wiles and Selker 2017) or H3K9me3 (Ninova *et al.* 2019), could in principle be incorporated in future versions to capture poised or repressive chromatin states not targeted here. As a deterministic rule-based method, REgulamentary emphasizes interpretability, but does not attempt to discover unanticipated or intermediate chromatin states, which probabilistic approaches are better suited to capture.

Firstly, it is widely known that directly measuring the activity of a *cis*-regulatory element is challenging, therefore it is common practice to infer the activity of an element based on chromatin accessibility, associated histone marks, and transcription factor binding. However, given the degree of overlap in these classifiers across Enhancers and Promoters, this is a complex task, and depending on the chosen method this can give varying results as we have demonstrated. It is known that specific compendiums of transcription factors bind at either Promoter or Enhancer elements, in a specific spatio-temporal manner. We therefore propose providing a list of ChIP-seq transcription factors peaks and intersecting these with the output annotations of REgulamentary to enhance the accuracy and biological significance of the output, by resolving the classifications for specific cell types or differentiation stages. In doing so the output of REgulamentary would have the ability to classify the regulatory elements by the activity of the elements, given transcription factor binding can be used as a proxy for this. Secondly, we would like to increase the speed and efficiency of the pipeline. The current version handles read counts, normalization, and *auc* computation in parallel using Python’s multiprocess feature, which despite being streamlined, still requires significant run-time (∼5 h) based on the size of the input data. To address this issue, we aim to implement Graphics Processing Units (GPUs) to increase the efficiency of data processing (Dally *et al.* 2021), and thereby speed up (∼10×) the assessment of *cis*-regulatory elements. Thirdly, even though we provided REgulamentary results for already 13 cell types, we aim to extend these by creating a robust and reliable roadmap of regulatory elements, covering as many cell types as possible. The boom in single-cell ATAC sequencing has created a wealth of genome accessibility data in hundreds of cell types, stages of development, and pathogenic contexts. REgulamentary has been shown to perform well with scATAC as input data (Gur and Hughes 2025), however, we intend to further develop REgulamentary to optimize specifically for this input.

This will create a first-in-class dataset which can be used to train, validate, and test Deep Learning (DL) Models (LeCun *et al.* 2015), such as Convolutional Neural Networks (O’Shea and Nash 2015), Recurrent Neural Networks (Medsker and Jain 1999), or Transformers-based networks (Wolf *et al.* 2020). This dataset will provide sufficient positive and validated examples of regulatory element regions per cell type in a suitable format for DL approaches, which will eliminate the need for histone marks and CTCF ChIP-seq input data, relying instead only on chromatin accessibility data to identify the different *cis*-regulatory elements accurately. Lastly, the developed front-end WebApp for REgulamentary output, made the presented tool more user-friendly and accessible to a broader range of scientists.

## Supplementary Material

vbag079_Supplementary_Data

## Data Availability

All data used for this work is publicly available. The list of data used for running REgulamentary on all 13 cell types is summarized in Table 5, available as supplementary data at *Bioinformatics Advances* online, whereas for the GWAS studies, we extracted them from the GWAS Catalogue (Sollis *et al.* 2023), and Table 4, available as supplementary data at *Bioinformatics Advances* online, reports detailed information about the used stroke variants. REgulamentary is a Python-based Snakemake (Mölder *et al.* 2021) pipeline built to identify *cis*-regulatory elements from sequenced data (GitHub: github.com/Genome-Function-Initiative-Oxford/REgulamentary), including instructions on how to create a project and how to view results by using its interactive visualization supported by MDV platform. The current public release of REgulamentary is stable and fully supports the analyses presented in this manuscript. Although the GitHub repository has not required recent changes, the tool continues to be actively maintained within our group. Planned updates include GPU acceleration, extended rule sets, simplified output options (e.g. automatic HTML or PDF summaries), and additional usability features, which will be incorporated into forthcoming releases.
