# Supplementary material for: Deciphering cis-regulatory elements using REgulamentary
Source: Bioinform Adv. 2026 Mar 20;6(1):vbag079. doi: 10.1093/bioadv/vbag079 (PMC13050536; doi:10.1093/bioadv/vbag079)
Supplement: vbag079_Supplementary_Data [file vbag079_supplementary_data.pdf]

Supplementary Figures

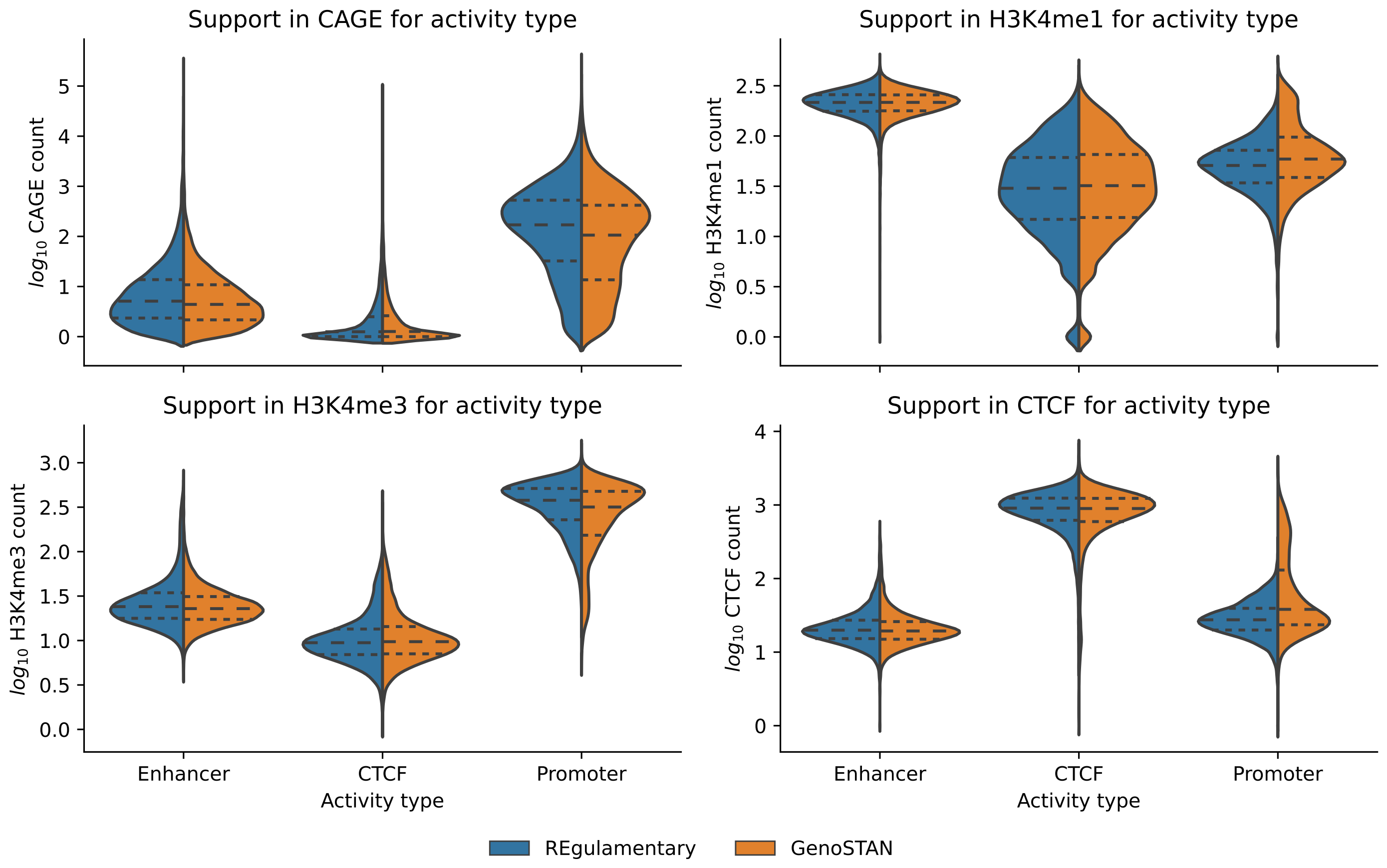

Supplementary Figure 1. Similar to Figure 4d in the main text, instead of distance to RefSeq TSS, we compute log_{10}(read-counts) for different experiment classes. We observe many GenoSTAN defined Promoters have high H3K4me1 counts, many have low H3K4me3 counts, many have low CAGE counts, and many have high CTCF ChIP counts. We observe a similar performance when compared to PolyA- and PolyA+ experiments.

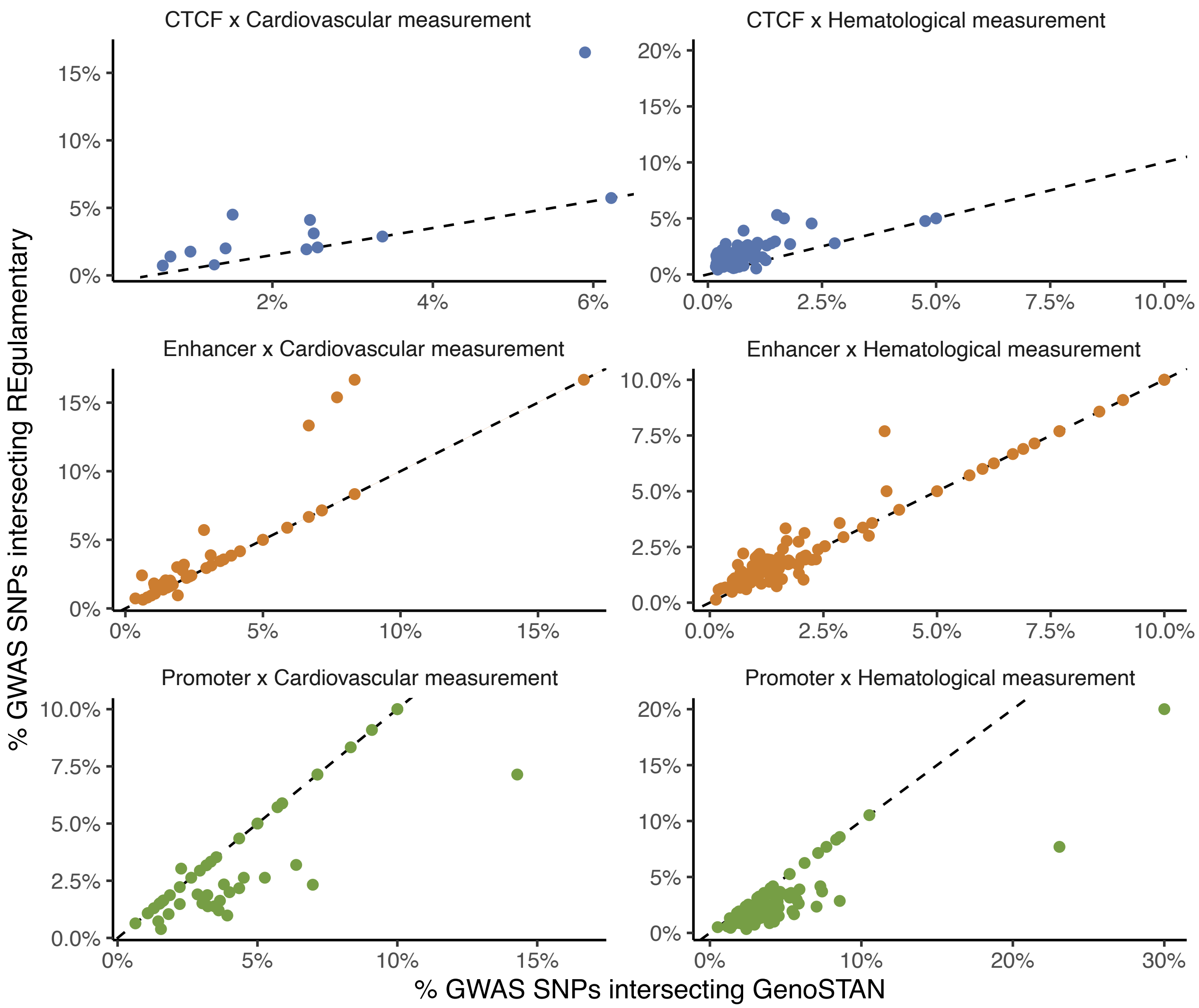

Supplementary Figure 2. Comparison of proportion of GWAS lead variants intersecting CTCF (top), Enhancer (middle), and Promoter (bottom) elements annotated by REgulamentary and GenoSTAN. Each point represents a different study of cardiovascular measurements (left) or hematological measurements (right) with at least 10 lead variants reported in GWAS Catalog version $1.0.2$ (downloaded on $22/08/2023$). GWASs were annotated to categories based on EFO mappings. The dotted line is the identity x=y.

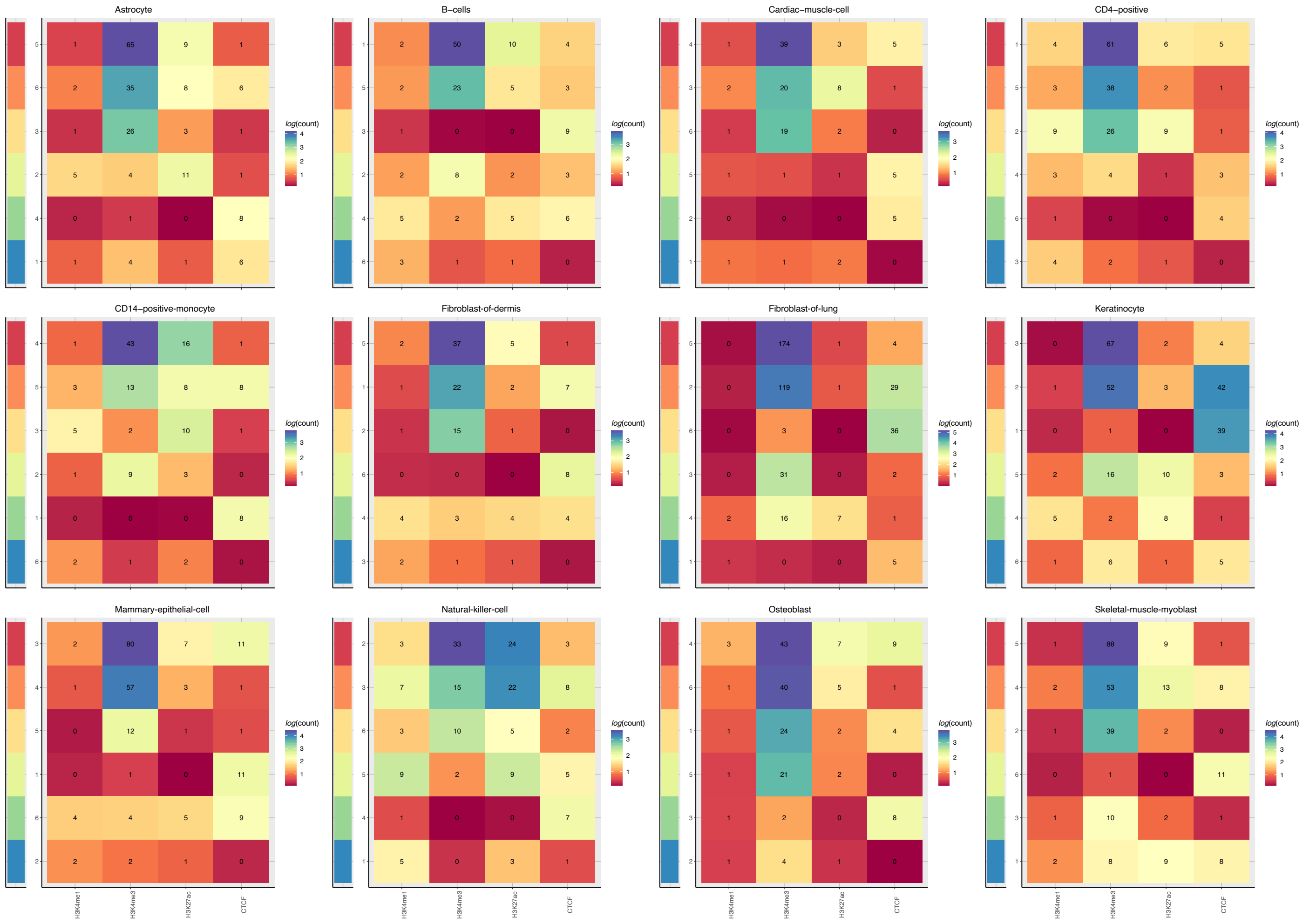

Supplementary Figure 3. log count of the median read coverage in GenoSTAN chromatin states per cell type, used to manually annotate each state in each cell type.

Supplementary Tables

Package/Library	Version
BEDtools	2.31.0
deepTools	3.5.2
LanceOtron	1.0.8
Matplotlib-base	3.8.0
NumPy	1.23.5
Pandas	2.1.1
pybedtools	0.9.1
pyBigWig	0.3.18
Samtools	1.16.1
scikit-learn	1.3.0
Seaborn	0.12.2

Supplementary Table 1. List of Python packages/libraries and their versions.

Cell type	Genomic feature	ENCODE Accession ID
HUVEC	DNase	ENCFF000STF
HUVEC	DNase	ENCFF000STI
HUVEC	DNase	ENCFF001BNW
HUVEC	DNase	ENCFF001BNX
HUVEC	DNase	ENCFF001BNY
HUVEC	DNase	ENCFF001BNZ
HUVEC	DNase	ENCFF001BOA
HUVEC	DNase	ENCFF001BOB
HUVEC	DNase	ENCFF001DOK
HUVEC	DNase	ENCFF001DOL
HUVEC	DNase	ENCFF044ZWE
HUVEC	DNase	ENCFF335SBI
HUVEC	DNase	ENCFF632YPM
HUVEC	DNase	ENCFF665FQP
HUVEC	H3K4me1	ENCFF000BSX
HUVEC	H3K4me1	ENCFF000BSY
HUVEC	H3K4me1	ENCFF000BTD
HUVEC	H3K4me3	ENCFF000BTH
HUVEC	H3K4me3	ENCFF000BTL
HUVEC	H3K4me3	ENCFF000BTS
HUVEC	H3K4me3	ENCFF001FSU
HUVEC	H3K4me3	ENCFF001FSV
HUVEC	H3K4me3	ENCFF255MRT
HUVEC	H3K4me3	ENCFF356SYW
HUVEC	H3K27ac	ENCFF000BSD
HUVEC	H3K27ac	ENCFF000BSI
HUVEC	CTCF	ENCFF000BQY
HUVEC	CTCF	ENCFF000BRD
HUVEC	CTCF	ENCFF000BRG
HUVEC	CTCF	ENCFF000RVE
HUVEC	CTCF	ENCFF000RVI
HUVEC	CTCF	ENCFF001HSL
HUVEC	CTCF	ENCFF001HSN

Supplementary Table 2. List of HUVEC type, genomic feature, and ENCODE Accession IDs.

Cell type	Genomic feature	ENCODE Accession ID
HUVEC	CAGE	ENCFF000UIX
HUVEC	CAGE	ENCFF000UJA
HUVEC	CAGE	ENCFF000UJF
HUVEC	CAGE	ENCFF000UJN
HUVEC	CAGE	ENCFF000UJO
HUVEC	CAGE	ENCFF000UJY
HUVEC	CAGE	ENCFF000UKB
HUVEC	CAGE	ENCFF000UKQ
HUVEC	CAGE	ENCFF000UKT

Supplementary Table 3. List of the 9 HUVEC CAGE data with their ENCODE Accession IDs.

Author	Published date	Study ID	Author	Published date	Study ID	Author	Published date	Study ID
Arning A	20/09/2012	GCST001689	Malik R	12/03/2018	GCST005842	Temprano-Sagrera G	14/03/2022	GCST90129551
Aung N	13/06/2022	GCST90134590	Malik R	12/03/2018	GCST005843	Temprano-Sagrera G	14/03/2022	GCST90129552
Aung N	13/06/2022	GCST90134594	Malik R	12/03/2018	GCST006906	Temprano-Sagrera G	14/03/2022	GCST90129553
Bellenguez C	05/02/2012	GCST001400	Malik R	12/03/2018	GCST006907	Temprano-Sagrera G	14/03/2022	GCST90129554
Carcel-Marquez J	08/07/2022	GCST90132228	Malik R	12/03/2018	GCST006908	Temprano-Sagrera G	14/03/2022	GCST90129555
Carrera C	16/03/2021	GCST90027882	Malik R	12/03/2018	GCST006909	Torres-Aguila NP	07/10/2019	GCST008915
Carty CL	18/06/2015	GCST002987	Malik R	12/03/2018	GCST006910	Traylor M	05/10/2012	GCST001706
Carty CL	18/06/2015	GCST002988	Malik R	01/11/2018	GCST007248	Traylor M	16/12/2015	GCST003245
Chauhan G	07/04/2016	GCST003490	Matarin M	06/05/2007	GCST000032	Traylor M	23/02/2016	GCST003423
Chauhan G	07/04/2016	GCST003491	Mishra A	30/09/2022	GCST90104536	Traylor M	20/12/2016	GCST004020
Cheng YC	05/01/2016	GCST003321	Mishra A	30/09/2022	GCST90104537	Traylor M	20/12/2016	GCST004022
Cheng YC	05/01/2016	GCST003322	Mishra A	30/09/2022	GCST90104538	Traylor M	20/12/2016	GCST004023
Cheng YC	05/01/2016	GCST003324	Mishra A	30/09/2022	GCST90104541	Traylor M	25/03/2021	GCST90014122
Daghals I	01/02/2022	GCST90103795	Mishra A	30/09/2022	GCST90104542	Traylor M	25/03/2021	GCST90014123
Daghals I	01/02/2022	GCST90103796	Mishra A	30/09/2022	GCST90104543	Verma SS	30/05/2020	GCST010483
Dichgans M	21/11/2013	GCST002286	Mishra A	30/09/2022	GCST90104553	von Berg J	11/02/2020	GCST010153
Dichgans M	21/11/2013	GCST002287	Mishra A	30/09/2022	GCST90162543	von Berg J	11/02/2020	GCST010154
Dichgans M	21/11/2013	GCST002288	Mishra A	30/09/2022	GCST90162544	von Berg J	11/02/2020	GCST010156
Dichgans M	21/11/2013	GCST002290	Mishra A	30/09/2022	GCST90162545	von Berg J	11/02/2020	GCST010158
Gretarsdottir S	01/10/2008	GCST000239	Mishra A	30/09/2022	GCST90162546	von Berg J	11/02/2020	GCST010159
Holliday EG	02/09/2012	GCST001659	Mola-Caminal M	01/01/2019	GCST006935	von Berg J	11/02/2020	GCST010160
Ibanez L	25/02/2022	GCST90239734	Pulit SL	18/12/2015	GCST003257	von Berg J	11/02/2020	GCST010161
Ibanez L	25/02/2022	GCST90239735	Pulit SL	18/12/2015	GCST003258	von Berg J	11/02/2020	GCST010163
Ibanez L	25/02/2022	GCST90239736	Pulit SL	18/12/2015	GCST003259	von Berg J	11/02/2020	GCST010164
Ibanez L	25/02/2022	GCST90239737	Pulit SL	18/12/2015	GCST003260	von Berg J	11/02/2020	GCST010165
Ikram MA	15/04/2009	GCST000379	Pulit SL	18/12/2015	GCST003261	von Berg J	11/02/2020	GCST010166
Ishigaki K	08/06/2020	GCST90013688	Sakaue S	30/09/2021	GCST90018644	von Berg J	11/02/2020	GCST010167
Jaworek T	11/09/2020	GCST010652	Sakaue S	30/09/2021	GCST90018864	Vujkovic M	15/06/2020	GCST010550
Jaworek T	31/08/2022	GCST90188370	Schmidt AF	26/04/2023	GCST90268122	Westphal S	24/01/2019	GCST007977
Jaworek T	31/08/2022	GCST90188371	Schmidt AF	26/04/2023	GCST90268130	Williams SR	30/12/2015	GCST003211
Keene KL	06/08/2014	GCST002559	Soderholm M	22/02/2019	GCST007672	Williams SR	30/12/2015	GCST003212
Keene KL	22/07/2020	GCST011176	Soderholm M	22/02/2019	GCST007673	Williams SR	30/12/2015	GCST003213
Kumar A	24/05/2021	GCST90020240	Soderholm M	22/02/2019	GCST007674	Williams SR	30/12/2015	GCST003233
Kumar A	24/05/2021	GCST90020241	Soderholm M	22/02/2019	GCST007675	Williams SR	30/12/2015	GCST003234
Kumar A	24/05/2021	GCST90020242	Soderholm M	22/02/2019	GCST007676	Williams SR	30/12/2015	GCST003235
Lee TH	09/11/2017	GCST005057	Song Y	25/02/2021	GCST90014126	Williams SR	01/06/2017	GCST004598
Malik R	06/03/2017	GCST007991	Song Y	25/02/2021	GCST90014129	Xie Y	10/02/2023	GCST90271319
Malik R	12/03/2018	GCST005838	Song Y	25/02/2021	GCST90014132	Yamada Y	29/05/2018	GCST010836
Malik R	12/03/2018	GCST005840	Temprano-Sagrera G	14/03/2022	GCST90129549	Zhang F	11/11/2021	GCST90095484
Malik R	12/03/2018	GCST005841	Temprano-Sagrera G	14/03/2022	GCST90129550			

Supplementary Table 4. List of author, published date, and the study ID information of GWAS study used for TopLD imputation.

Cell type	Genomic feature	ENCODE Accession ID	Cell type	Genomic feature	ENCODE Accession ID
Astrocyte	DNase	ENCFF001CEM	B-cell	CTCF	ENCFF077FWS
Astrocyte	DNase	ENCFF001EBK	B-cell	CTCF	ENCFF410CFV
Astrocyte	DNase	ENCFF001EBL	B-cell	CTCF	ENCFF447FUF
Astrocyte	H3K4me1	ENCFF000CHZ	B-cell	CTCF	ENCFF455HJY
Astrocyte	H3K4me1	ENCFF000CIA	B-cell	CTCF	ENCFF528QNI
Astrocyte	H3K4me3	ENCFF000CID	B-cell	CTCF	ENCFF538KCH
Astrocyte	H3K4me3	ENCFF000CIG	B-cell	CTCF	ENCFF641ERL
Astrocyte	H3K27ac	ENCFF000CHH	B-cell	CTCF	ENCFF657DUW
Astrocyte	H3K27ac	ENCFF000CHK	B-cell	CTCF	ENCFF674DJW
Astrocyte	CTCF	ENCFF000CFV	B-cell	CTCF	ENCFF866NIM
Astrocyte	CTCF	ENCFF000CFW	CD14-positive-monocyte	DNase	ENCFF000TBL
Astrocyte	CTCF	ENCFF059YWU	CD14-positive-monocyte	DNase	ENCFF000TBP
Astrocyte	CTCF	ENCFF198SPA	CD14-positive-monocyte	DNase	ENCFF001DZG
Astrocyte	CTCF	ENCFF426NZW	CD14-positive-monocyte	DNase	ENCFF001DZH
Astrocyte	CTCF	ENCFF514PBH	CD14-positive-monocyte	DNase	ENCFF231SIM
Astrocyte	CTCF	ENCFF545OBJ	CD14-positive-monocyte	DNase	ENCFF250JVY
Astrocyte	CTCF	ENCFF617KMB	CD14-positive-monocyte	DNase	ENCFF470FVJ
Astrocyte	CTCF	ENCFF635WSD	CD14-positive-monocyte	DNase	ENCFF690LNW
Astrocyte	CTCF	ENCFF641PTH	CD14-positive-monocyte	DNase	ENCFF971DJA
B-cell	DNase	ENCFF000SFP	CD14-positive-monocyte	H3K4me1	ENCFF000CDK
B-cell	DNase	ENCFF000SFS	CD14-positive-monocyte	H3K4me1	ENCFF000CDL
B-cell	DNase	ENCFF001CPI	CD14-positive-monocyte	H3K4me1	ENCFF076WOE
B-cell	DNase	ENCFF001CQF	CD14-positive-monocyte	H3K4me1	ENCFF402BCW
B-cell	DNase	ENCFF001CQG	CD14-positive-monocyte	H3K4me1	ENCFF858VSO
B-cell	DNase	ENCFF064WNA	CD14-positive-monocyte	H3K4me3	ENCFF000CDX
B-cell	DNase	ENCFF122YDK	CD14-positive-monocyte	H3K4me3	ENCFF000CDZ
B-cell	DNase	ENCFF336XNG	CD14-positive-monocyte	H3K4me3	ENCFF001FYS
B-cell	DNase	ENCFF425RAB	CD14-positive-monocyte	H3K4me3	ENCFF399MSH
B-cell	DNase	ENCFF431JPD	CD14-positive-monocyte	H3K4me3	ENCFF820FZP
B-cell	DNase	ENCFF519OTF	CD14-positive-monocyte	H3K4me3	ENCFF992EVR
B-cell	DNase	ENCFF528MVU	CD14-positive-monocyte	H3K27ac	ENCFF000CEN
B-cell	DNase	ENCFF541ZNP	CD14-positive-monocyte	H3K27ac	ENCFF000CEO
B-cell	DNase	ENCFF566GSP	CD14-positive-monocyte	H3K27ac	ENCFF511CWO
B-cell	DNase	ENCFF666GUI	CD14-positive-monocyte	H3K27ac	ENCFF765EVS
B-cell	DNase	ENCFF759SXV	CD14-positive-monocyte	H3K27ac	ENCLB008QBO
B-cell	H3K4me1	ENCFF031DVC	CD14-positive-monocyte	H3K27ac	ENCLB008QBO
B-cell	H3K4me1	ENCFF107GUJ	CD14-positive-monocyte	H3K27ac	ENCLB709FWW
B-cell	H3K4me1	ENCFF474DOG	CD14-positive-monocyte	H3K27ac	ENCLB709FWW
B-cell	H3K4me1	ENCFF653MQR	CD14-positive-monocyte	CTCF	ENCFF000CCY
B-cell	H3K4me1	ENCFF944BAK	CD14-positive-monocyte	CTCF	ENCFF000CCZ
B-cell	H3K4me1	ENCFF968ZDD	CD14-positive-monocyte	CTCF	ENCFF021GJE
B-cell	H3K4me3	ENCFF001ETL	CD14-positive-monocyte	CTCF	ENCFF023DJQ
B-cell	H3K4me3	ENCFF001ETS	CD14-positive-monocyte	CTCF	ENCFF047VHU
B-cell	H3K4me3	ENCFF001EUE	CD14-positive-monocyte	CTCF	ENCFF197MJW
B-cell	H3K4me3	ENCFF136DRG	CD14-positive-monocyte	CTCF	ENCFF278JEO
B-cell	H3K4me3	ENCFF329DZQ	CD14-positive-monocyte	CTCF	ENCFF435DNT
B-cell	H3K4me3	ENCFF527EXT	CD14-positive-monocyte	CTCF	ENCFF504EHB
B-cell	H3K4me3	ENCFF659TTV	CD14-positive-monocyte	CTCF	ENCFF701WZE
B-cell	H3K4me3	ENCFF715ZJM	CD14-positive-monocyte	CTCF	ENCFF763QZI
B-cell	H3K4me3	ENCFF751FJK	CD14-positive-monocyte	CTCF	ENCFF777QSB
B-cell	H3K4me3	ENCFF761FXO	CD14-positive-monocyte	CTCF	ENCFF892PTF
B-cell	H3K4me3	ENCFF927VYA	CD14-positive-monocyte	CTCF	ENCFF893PQO
B-cell	H3K27ac	ENCFF000ANT	Cardiac-muscle-cell	DNase	ENCFF001BAK
B-cell	H3K27ac	ENCFF000ANV	Cardiac-muscle-cell	DNase	ENCFF001BAL
B-cell	H3K27ac	ENCFF861JPC	Cardiac-muscle-cell	DNase	ENCFF001BAM
B-cell	H3K27ac	ENCFF995OBQ	Cardiac-muscle-cell	DNase	ENCFF001DBE
B-cell	CTCF	ENCFF000AMR	Cardiac-muscle-cell	DNase	ENCFF001DBH
B-cell	CTCF	ENCFF000AMS	Cardiac-muscle-cell	DNase	ENCFF046CJQ
B-cell	CTCF	ENCFF000AMU	Cardiac-muscle-cell	DNase	ENCFF529GIA
B-cell	CTCF	ENCFF039AQL	Cardiac-muscle-cell	DNase	ENCFF781FBG
B-cell	CTCF	ENCFF071JZD	Cardiac-muscle-cell	H3K4me1	ENCFF002NBE

Cell type	Genomic feature	ENCODE Accession ID	Cell type	Genomic feature	ENCODE Accession ID
Cardiac-muscle-cell	H3K4me1	ENCFF024XLI	CD4-positive_alpha-beta-t-cell	H3K4me1	ENCFF346CXE
Cardiac-muscle-cell	H3K4me1	ENCFF044YWU	CD4-positive_alpha-beta-t-cell	H3K4me1	ENCFF609VUZ
Cardiac-muscle-cell	H3K4me1	ENCFF078OYE	CD4-positive_alpha-beta-t-cell	H3K4me3	ENCFF051MYP
Cardiac-muscle-cell	H3K4me1	ENCFF109CIF	CD4-positive_alpha-beta-t-cell	H3K4me3	ENCFF124FAJ
Cardiac-muscle-cell	H3K4me1	ENCFF523UYJ	CD4-positive_alpha-beta-t-cell	H3K4me3	ENCFF534ZEZ
Cardiac-muscle-cell	H3K4me1	ENCFF664TIA	CD4-positive_alpha-beta-t-cell	H3K4me3	ENCFF546HLT
Cardiac-muscle-cell	H3K4me1	ENCFF738LVV	CD4-positive_alpha-beta-t-cell	H3K4me3	ENCFF968VEH
Cardiac-muscle-cell	H3K4me1	ENCFF744FRZ	CD4-positive_alpha-beta-t-cell	H3K4me3	ENCFF992ODQ
Cardiac-muscle-cell	H3K4me1	ENCFF784FGJ	CD4-positive_alpha-beta-t-cell	H3K27ac	ENCFF017MGJ
Cardiac-muscle-cell	H3K4me1	ENCFF794RCZ	CD4-positive_alpha-beta-t-cell	H3K27ac	ENCFF416ZFL
Cardiac-muscle-cell	H3K4me1	ENCFF825YRT	CD4-positive_alpha-beta-t-cell	CTCF	ENCFF093IMN
Cardiac-muscle-cell	H3K4me1	ENCFF884AZS	CD4-positive_alpha-beta-t-cell	CTCF	ENCFF105VUO
Cardiac-muscle-cell	H3K4me1	ENCFF928UVS	CD4-positive_alpha-beta-t-cell	CTCF	ENCFF134YVA
Cardiac-muscle-cell	H3K4me1	ENCFF943GKJ	CD4-positive_alpha-beta-t-cell	CTCF	ENCFF267UOX
Cardiac-muscle-cell	H3K4me1	ENCFF962AHN	CD4-positive_alpha-beta-t-cell	CTCF	ENCFF271IZY
Cardiac-muscle-cell	H3K4me3	ENCFF001FHR	CD4-positive_alpha-beta-t-cell	CTCF	ENCFF382ACD
Cardiac-muscle-cell	H3K4me3	ENCFF001FIA	CD4-positive_alpha-beta-t-cell	CTCF	ENCFF549TBL
Cardiac-muscle-cell	H3K4me3	ENCFF055SGG	CD4-positive_alpha-beta-t-cell	CTCF	ENCFF577GDR
Cardiac-muscle-cell	H3K4me3	ENCFF136HNS	CD4-positive_alpha-beta-t-cell	CTCF	ENCFF609XGR
Cardiac-muscle-cell	H3K4me3	ENCFF161RBU	CD4-positive_alpha-beta-t-cell	CTCF	ENCFF683UCV
Cardiac-muscle-cell	H3K4me3	ENCFF204HFJ	CD4-positive_alpha-beta-t-cell	CTCF	ENCFF696VYZ
Cardiac-muscle-cell	H3K4me3	ENCFF430BVZ	CD4-positive_alpha-beta-t-cell	CTCF	ENCFF812FXF
Cardiac-muscle-cell	H3K4me3	ENCFF712AIJ	Fibroblast-of-dermis	DNase	ENCFF001CES
Cardiac-muscle-cell	H3K4me3	ENCFF823MWD	Fibroblast-of-dermis	DNase	ENCFF001ECC
Cardiac-muscle-cell	H3K4me3	ENCFF881COR	Fibroblast-of-dermis	DNase	ENCFF001ECD
Cardiac-muscle-cell	H3K4me3	ENCFF911NYD	Fibroblast-of-dermis	H3K4me1	ENCFF000CJT
Cardiac-muscle-cell	H3K4me3	ENCFF989CNP	Fibroblast-of-dermis	H3K4me1	ENCFF000CJY
Cardiac-muscle-cell	H3K27ac	ENCFF143SXY	Fibroblast-of-dermis	H3K4me3	ENCFF000CLE
Cardiac-muscle-cell	H3K27ac	ENCFF232TCS	Fibroblast-of-dermis	H3K4me3	ENCFF000CLG
Cardiac-muscle-cell	H3K27ac	ENCFF236ZHP	Fibroblast-of-dermis	H3K4me3	ENCFF170GWK
Cardiac-muscle-cell	H3K27ac	ENCFF299TZK	Fibroblast-of-dermis	H3K4me3	ENCFF217GRK
Cardiac-muscle-cell	H3K27ac	ENCFF340KQN	Fibroblast-of-dermis	H3K27ac	ENCFF000CKG
Cardiac-muscle-cell	H3K27ac	ENCFF366LCI	Fibroblast-of-dermis	H3K27ac	ENCFF000CKH
Cardiac-muscle-cell	H3K27ac	ENCFF402UQN	Fibroblast-of-dermis	CTCF	ENCFF000CJC
Cardiac-muscle-cell	H3K27ac	ENCFF478OZK	Fibroblast-of-dermis	CTCF	ENCFF000CJD
Cardiac-muscle-cell	H3K27ac	ENCFF577YKS	Fibroblast-of-lung	DNase	ENCFF001BQM
Cardiac-muscle-cell	H3K27ac	ENCFF766VGS	Fibroblast-of-lung	DNase	ENCFF001CBY
Cardiac-muscle-cell	CTCF	ENCFF001HKU	Fibroblast-of-lung	DNase	ENCFF001CBZ
Cardiac-muscle-cell	CTCF	ENCFF001HKV	Fibroblast-of-lung	DNase	ENCFF001CCA
Cardiac-muscle-cell	CTCF	ENCFF096TTP	Fibroblast-of-lung	DNase	ENCFF001DLE
Cardiac-muscle-cell	CTCF	ENCFF388XOP	Fibroblast-of-lung	DNase	ENCFF001DLF
Cardiac-muscle-cell	CTCF	ENCFF562AGE	Fibroblast-of-lung	DNase	ENCFF001EDI
Cardiac-muscle-cell	CTCF	ENCFF689PWV	Fibroblast-of-lung	DNase	ENCFF001EDJ
CD4-positive_alpha-beta-t-cell	DNase	ENCFF041CSN	Fibroblast-of-lung	DNase	ENCFF071BZF
CD4-positive_alpha-beta-t-cell	DNase	ENCFF083PGI	Fibroblast-of-lung	DNase	ENCFF133ACJ
CD4-positive_alpha-beta-t-cell	DNase	ENCFF116MII	Fibroblast-of-lung	DNase	ENCFF628VTP
CD4-positive_alpha-beta-t-cell	DNase	ENCFF169NOQ	Fibroblast-of-lung	DNase	ENCFF913UMD
CD4-positive_alpha-beta-t-cell	DNase	ENCFF212JQA	Fibroblast-of-lung	H3K4me1	ENCFF000CSJ
CD4-positive_alpha-beta-t-cell	DNase	ENCFF263GAW	Fibroblast-of-lung	H3K4me1	ENCFF000CSM
CD4-positive_alpha-beta-t-cell	DNase	ENCFF359YML	Fibroblast-of-lung	H3K4me3	ENCFF000CST
CD4-positive_alpha-beta-t-cell	DNase	ENCFF388MZH	Fibroblast-of-lung	H3K4me3	ENCFF000CSY
CD4-positive_alpha-beta-t-cell	DNase	ENCFF412KYG	Fibroblast-of-lung	H3K4me3	ENCFF001FPZ
CD4-positive_alpha-beta-t-cell	DNase	ENCFF802YCL	Fibroblast-of-lung	H3K4me3	ENCFF001FQA
CD4-positive_alpha-beta-t-cell	DNase	ENCFF862RQS	Fibroblast-of-lung	H3K4me3	ENCFF001GBC
CD4-positive_alpha-beta-t-cell	DNase	ENCFF879IJW	Fibroblast-of-lung	H3K4me3	ENCFF001GBF
CD4-positive_alpha-beta-t-cell	DNase	ENCFF931EXL	Fibroblast-of-lung	H3K4me3	ENCFF216QPB
CD4-positive_alpha-beta-t-cell	DNase	ENCFF947ORJ	Fibroblast-of-lung	H3K4me3	ENCFF238HDJ
CD4-positive_alpha-beta-t-cell	H3K4me1	ENCFF096ADV	Fibroblast-of-lung	H3K4me3	ENCFF421EYR
CD4-positive_alpha-beta-t-cell	H3K4me1	ENCFF167HLM	Fibroblast-of-lung	H3K4me3	ENCFF612NOM
CD4-positive_alpha-beta-t-cell	H3K4me1	ENCFF284RQL	Fibroblast-of-lung	H3K27ac	ENCFF000CRS
CD4-positive_alpha-beta-t-cell	H3K4me1	ENCFF312WMK	Fibroblast-of-lung	H3K27ac	ENCFF000CRV

Cell type	Genomic feature	ENCODE Accession ID	Cell type	Genomic feature	ENCODE Accession ID
Fibroblast-of-lung	CTCF	ENCFF000CQU	Natural-killer-cell	DNase	ENCFF431SXN
Fibroblast-of-lung	CTCF	ENCFF000CQV	Natural-killer-cell	DNase	ENCFF505OFY
Fibroblast-of-lung	CTCF	ENCFF001HQZ	Natural-killer-cell	DNase	ENCFF521XLX
Fibroblast-of-lung	CTCF	ENCFF001HRA	Natural-killer-cell	DNase	ENCFF721PPR
Fibroblast-of-lung	CTCF	ENCFF001HWA	Natural-killer-cell	DNase	ENCFF773JXH
Keratinocyte	DNase	ENCFF000TBS	Natural-killer-cell	DNase	ENCFF809SDD
Keratinocyte	DNase	ENCFF000TBT	Natural-killer-cell	H3K4me1	ENCFF023WJX
Keratinocyte	DNase	ENCFF001ECW	Natural-killer-cell	H3K4me1	ENCFF107VGZ
Keratinocyte	DNase	ENCFF001ECX	Natural-killer-cell	H3K4me3	ENCFF573HXM
Keratinocyte	H3K4me1	ENCFF000COE	Natural-killer-cell	H3K4me3	ENCFF679OKE
Keratinocyte	H3K4me1	ENCFF000COI	Natural-killer-cell	H3K27ac	ENCFF002LAJ
Keratinocyte	H3K4me1	ENCFF000COL	Natural-killer-cell	H3K27ac	ENCFF612WYS
Keratinocyte	H3K4me3	ENCFF000COU	Natural-killer-cell	CTCF	ENCFF036MEY
Keratinocyte	H3K4me3	ENCFF000COV	Natural-killer-cell	CTCF	ENCFF252VPJ
Keratinocyte	H3K4me3	ENCFF000CPA	Natural-killer-cell	CTCF	ENCFF286OQT
Keratinocyte	H3K4me3	ENCFF001GAN	Natural-killer-cell	CTCF	ENCFF562YWJ
Keratinocyte	H3K4me3	ENCFF001GAV	Natural-killer-cell	CTCF	ENCFF634FNO
Keratinocyte	H3K4me3	ENCFF259YKX	Natural-killer-cell	CTCF	ENCFF647URX
Keratinocyte	H3K4me3	ENCFF300OUH	Natural-killer-cell	CTCF	ENCFF659OED
Keratinocyte	H3K4me3	ENCFF774JBP	Natural-killer-cell	CTCF	ENCFF667ISZ
Keratinocyte	H3K27ac	ENCFF000CNL	Natural-killer-cell	CTCF	ENCFF775YPL
Keratinocyte	H3K27ac	ENCFF000CNN	Natural-killer-cell	CTCF	ENCFF854KQD
Keratinocyte	CTCF	ENCFF000CMF	Natural-killer-cell	CTCF	ENCFF931CRX
Keratinocyte	CTCF	ENCFF000CMG	Natural-killer-cell	CTCF	ENCFF945IEZ
Keratinocyte	CTCF	ENCFF000CMM	Osteoblast	DNase	ENCFF000TCL
Keratinocyte	CTCF	ENCFF000SCQ	Osteoblast	DNase	ENCFF000TCM
Keratinocyte	CTCF	ENCFF000SCV	Osteoblast	DNase	ENCFF000TCW
Keratinocyte	CTCF	ENCFF001HVN	Osteoblast	H3K4me1	ENCFF000CWD
Keratinocyte	CTCF	ENCFF001HVQ	Osteoblast	H3K4me1	ENCFF000CWF
Mammary-epithelial-cell	DNase	ENCFF001DFW	Osteoblast	H3K4me3	ENCFF000CTU
Mammary-epithelial-cell	DNase	ENCFF001DFX	Osteoblast	H3K4me3	ENCFF000CTV
Mammary-epithelial-cell	DNase	ENCFF040XDV	Osteoblast	H3K4me3	ENCFF000CTW
Mammary-epithelial-cell	DNase	ENCFF289HNI	Osteoblast	H3K4me3	ENCFF671ROB
Mammary-epithelial-cell	DNase	ENCFF356XGS	Osteoblast	H3K4me3	ENCFF769IGM
Mammary-epithelial-cell	DNase	ENCFF426PKC	Osteoblast	H3K27ac	ENCFF000CVS
Mammary-epithelial-cell	DNase	ENCFF464VLQ	Osteoblast	H3K27ac	ENCFF000CVU
Mammary-epithelial-cell	DNase	ENCFF660OYN	Osteoblast	CTCF	ENCFF000CVF
Mammary-epithelial-cell	DNase	ENCFF726SZK	Osteoblast	CTCF	ENCFF000CVG
Mammary-epithelial-cell	DNase	ENCFF914GIZ	Skeletal-muscle-myoblast	DNase	ENCFF001DNY
Mammary-epithelial-cell	H3K4me1	ENCFF000BJD	Skeletal-muscle-myoblast	DNase	ENCFF001DNZ
Mammary-epithelial-cell	H3K4me1	ENCFF000BJE	Skeletal-muscle-myoblast	DNase	ENCFF018BZJ
Mammary-epithelial-cell	H3K4me3	ENCFF000BJN	Skeletal-muscle-myoblast	DNase	ENCFF218GIV
Mammary-epithelial-cell	H3K4me3	ENCFF000BJO	Skeletal-muscle-myoblast	DNase	ENCFF337WYN
Mammary-epithelial-cell	H3K4me3	ENCFF001FOG	Skeletal-muscle-myoblast	DNase	ENCFF389LSY
Mammary-epithelial-cell	H3K4me3	ENCFF001FOH	Skeletal-muscle-myoblast	DNase	ENCFF404QTI
Mammary-epithelial-cell	H3K4me3	ENCFF113ECY	Skeletal-muscle-myoblast	DNase	ENCFF418ZDR
Mammary-epithelial-cell	H3K4me3	ENCFF174YML	Skeletal-muscle-myoblast	DNase	ENCFF465ATZ
Mammary-epithelial-cell	H3K4me3	ENCFF240ZWX	Skeletal-muscle-myoblast	DNase	ENCFF689CAW
Mammary-epithelial-cell	H3K4me3	ENCFF352FFB	Skeletal-muscle-myoblast	DNase	ENCFF845QRZ
Mammary-epithelial-cell	H3K4me3	ENCFF904QEC	Skeletal-muscle-myoblast	DNase	ENCFF875UUR
Mammary-epithelial-cell	H3K27ac	ENCFF000BIL	Skeletal-muscle-myoblast	H3K4me1	ENCFF000BLX
Mammary-epithelial-cell	H3K27ac	ENCFF000BIM	Skeletal-muscle-myoblast	H3K4me1	ENCFF000BMA
Mammary-epithelial-cell	CTCF	ENCFF000BHI	Skeletal-muscle-myoblast	H3K4me3	ENCFF000BML
Mammary-epithelial-cell	CTCF	ENCFF000BHL	Skeletal-muscle-myoblast	H3K4me3	ENCFF000BMP
Mammary-epithelial-cell	CTCF	ENCFF001HPL	Skeletal-muscle-myoblast	H3K4me3	ENCFF102BFB
Mammary-epithelial-cell	CTCF	ENCFF001HPM	Skeletal-muscle-myoblast	H3K4me3	ENCFF243OFA
Natural-killer-cell	DNase	ENCFF043NIU	Skeletal-muscle-myoblast	H3K4me3	ENCFF794IID
Natural-killer-cell	DNase	ENCFF137TAG	Skeletal-muscle-myoblast	H3K27ac	ENCFF000BLI
Natural-killer-cell	DNase	ENCFF215HOZ	Skeletal-muscle-myoblast	H3K27ac	ENCFF000BLJ
Natural-killer-cell	DNase	ENCFF384FJC	Skeletal-muscle-myoblast	CTCF	ENCFF000BKP
Natural-killer-cell	DNase	ENCFF423GQM	Skeletal-muscle-myoblast	CTCF	ENCFF000BKS

Supplementary Table 5. List of the 12 cell type, genomic feature, and ENCODE Accession IDs.

Cell type	State 1	State 2	State 3	State 4	State 5	State 6
Astrocyte	Promoter/CTCF	*	Promoter	CTCF	Promoter	Promoter
B-cell	Promoter	Promoter	CTCF	Enhancer/CTCF	Promoter	Enhancer
Cardiac-muscle-cell	*	CTCF	Promoter	Promoter	CTCF	Promoter
CD4-positive_alpha-beta-t-cell	Promoter	Promoter	Enhancer	*	Promoter	CTCF
CD14-positive-monocyte	CTCF	Promoter	Enhancer	Promoter	Promoter	*
Fibroblast-of-dermis	Promoter	Promoter	*	*	Promoter	CTCF
Fibroblast-of-lung	CTCF	Promoter	Promoter	Promoter	Promoter	CTCF
Keratinocyte	CTCF	Promoter	Promoter	Enhancer	Promoter	Promoter
Mammary-epithelial-cell	CTCF	*	Promoter	Promoter	Promoter	CTCF
Natural-killer-cell	Enhancer	Promoter	Promoter	CTCF	Enhancer	Promoter
Osteoblast	Promoter	Promoter	CTCF	Promoter	Promoter	Promoter
Skeletal-muscle-myoblas	Promoter/CTCF	Promoter	Promoter	Promoter	Promoter	CTCF

Supplementary Table 6. Manual assessment of GenoSTAN states, based on log scaled read counts (Supplementary Figure 3), according to the REgulamentary naming assignment. The asterisk ($*$) means that the GenoSTAN state is unclear.

Cell Type	MDV Project URL
B-cell	`https://mdv.molbiol.ox.ac.uk/projects/mdv_project/7586`
Cardiac muscle cell	`https://mdv.molbiol.ox.ac.uk/projects/mdv_project/7587`
Endothelial cell of umbilical vein	`https://mdv.molbiol.ox.ac.uk/projects/mdv_project/7588`
Keratinocyte	`https://mdv.molbiol.ox.ac.uk/projects/mdv_project/7589`
Natural killer cell	`https://mdv.molbiol.ox.ac.uk/projects/mdv_project/7590`
CD4-positive T cell	`https://mdv.molbiol.ox.ac.uk/projects/mdv_project/7591`
Fibroblast of lung	`https://mdv.molbiol.ox.ac.uk/projects/mdv_project/7592`
CD14-positive monocyte	`https://mdv.molbiol.ox.ac.uk/projects/mdv_project/7593`
Fibroblast of dermis	`https://mdv.molbiol.ox.ac.uk/projects/mdv_project/7594`
Osteoblast	`https://mdv.molbiol.ox.ac.uk/projects/mdv_project/7595`
Skeletal muscle myoblast	`https://mdv.molbiol.ox.ac.uk/projects/mdv_project/7596`
Mammary epithelial cell	`https://mdv.molbiol.ox.ac.uk/projects/mdv_project/7597`
Astrocyte	`https://mdv.molbiol.ox.ac.uk/projects/mdv_project/7598`

Supplementary Table 7. MDV interactive visualisation projects for the 12 additional ENCODE cell types.
